# An algorithm for learning shape and appearance models without annotations

**DOI:** 10.1016/j.media.2019.04.008

**Published:** 2019-07

**Authors:** John Ashburner, Mikael Brudfors, Kevin Bronik, Yaël Balbastre

**Affiliations:** Wellcome Centre for Human NeuroimagingUCL Queen Square Institute of Neurology12 Queen Square, London, WC1N 3AR, UK

**Keywords:** Machine learning, Latent variables, Diffeomorphisms, Geodesic shooting, Shape model, Appearance model

## Abstract

This paper presents a framework for automatically learning shape and appearance models for medical (and certain other) images. The algorithm was developed with the aim of eventually enabling distributed privacy-preserving analysis of brain image data, such that shared information (shape and appearance basis functions) may be passed across sites, whereas latent variables that encode individual images remain secure within each site. These latent variables are proposed as features for privacy-preserving data mining applications.

The approach is demonstrated qualitatively on the KDEF dataset of 2D face images, showing that it can align images that traditionally require shape and appearance models trained using manually annotated data (manually defined landmarks etc.). It is applied to the MNIST dataset of handwritten digits to show its potential for machine learning applications, particularly when training data is limited. The model is able to handle “missing data”, which allows it to be cross-validated according to how well it can predict left-out voxels. The suitability of the derived features for classifying individuals into patient groups was assessed by applying it to a dataset of over 1900 segmented T1-weighted MR images, which included images from the COBRE and ABIDE datasets.

## Highlights

• A framework for automatically learning shape and appearance models without manual annotations.
• Designed to run within a distributed privacy preserving framework.
• When used as a pattern recognition approach, can give competitive classification accuracies for MNIST - particularly for small numbers of training examples.
• Can handle missing data in the images.
• Tested the model with 1900 brain scans and found that its latent variables can be used as features for pattern recognition.

## Graphical abstract

**Figure:**
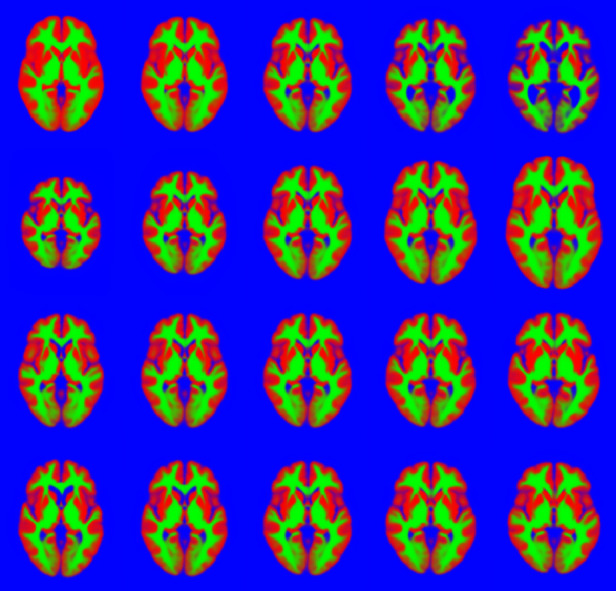

## 1 Introduction

This paper introduces an algorithm for learning a model of shape and appearance variability from a collection of images, without relying on manual annotations. The shape part of the model concerns modelling variability with diffeomorphic deformations, which is essentially image registration. In contrast, the appearance part is about accounting for signal variability that is not well described by deformations, and is essentially about adapting a “template” to enable more precise registration.

The problem of image registration is sometimes viewed from a Bayesian perspective, whereby the aim is to determine the most probable deformation (*ψ*) given the fixed (**f**) and moving (***μ***) images

(1) $$\hat{\psi }=\underset{\psi }{\arg\max}\log p\left(\psi |f,\mu \right)=\underset{\psi }{\arg\max}\left(\log p(f|\psi ,\mu )+\log p(\psi )\right).$$

In practice, the regularisation term (log *p*(*ψ*)) is not usually defined empirically, and simply involves a penalty based on some simple measure of deformation smoothness. One of the aims of this work is to try to improve on this simple model. By providing empirically derived priors for the allowable deformations, trained shape models have been shown to exhibit more robust image registration. An early example is Cootes and Taylor (1992), in which control point positions are constrained by their first few modes of variability. Training this model involved annotating images by manually placing a number of corresponding landmarks, computing the mean and covariance of the collection of landmarks, and then computing the eigenvectors of the covariance (Cootes et al., 1995). In neuroimaging, shape models have previously been used to increase the robustness of brain image segmentation (Babalola, Patenaude, Aljabar, Schnabel, Kennedy, Crum, Smith, Cootes, Jenkinson, Rueckert, 2009, Patenaude, Smith, Kennedy, Jenkinson, 2011). The current work involves densely parameterised shape models within the diffeomorphic setting, and relates to previous work on diffeomorphic shape models (Cootes et al., 2008), as well as those using more densely parameterised deformations (Rueckert et al., 2003). Recently, Zhang and Fletcher (2015) developed their Principal Geodesic Analysis (PGA) framework for directly computing the main modes of shape variation within a diffeomorphic setting.

In addition to increasing the robustness of image registration tasks, shape models can also provide features that may be used for statistical shape analysis. This is related to approaches used in geometric morphometrics (Adams et al., 2004), where the aim is to understand shape differences among anatomies. Shape descriptors from the PGA framework have previously been found to be useful features for data mining (Zhang et al., 2017).

A number of works have investigated combining both shape and appearance variability into the same model (Cootes, Taylor, Cooper, Graham, 1995, Cootes, Edwards, Taylor, 2001, Cootes, Taylor, 2001, Cootes, Twining, Babalola, Taylor, 2008, Belongie, Malik, Puzicha, 2002, Patenaude, Smith, Kennedy, Jenkinson, 2011). These combined shape and appearance models have generally shown good performance in a number of medical imaging challenges (Litjens et al., 2014). While there is quite a lot written about learning appearance variability alone, the literature on automatically learning both shape and appearance together is fairly limited. Earlier approaches required annotated data for training, but there are now some works appearing that have looked into the possibility of using unsupervised or semi-supervised approaches for learning shape and appearance variability. Examples include Cootes et al. (2010), Alabort-i Medina and Zafeiriou (2014), Lindner et al. (2015) and Štern et al. (2016). The current work is about an unsupervised approach, but there is no reason why it could not be made semi-supervised by also incorporating some manually defined landmarks or other features.

This work was undertaken as a task in the Medical Informatics Platform of the EU Human Brain Project (HBP). The original aim of the Medical Informatics Platform was to develop a distributed knowledge discovery framework that enables data mining without violating patient confidentiality. The strategy was to involve a horizontally partitioned dataset, where data about different patients is stored in different hospital sites. Although this has not been done, the algorithm presented in this paper can be implemented (see Section 2.2) in a way that does not require patient-specific information to leave a site, and instead only shares aggregates, which reveal less about the individual subjects. Some leakage of information (potentially exploitable by those with malicious intent) is inevitable, particularly for sites holding data on only small numbers of individuals, but we leave this as a topic to be addressed elsewhere. Aggregated data may be weighted moments (e.g. ∑_*n*_*r_n_*, ∑_*n*_*r_n_***z**_*n*_ or $\sum_{n}r_{n}z_{n}z_{n}^{T},$ where **z**_*n*_ is a vector of values for patient *n*, and *r_n_* is a patient-specific weight generated by some rule), which could then be used for clustering or other forms of statistical analysis. Enabling this type of approach to be applied to images requires some form of dimensionality reduction, particularly if covariances need to be represented (such as for clustering into patient subgroups using Gaussian mixture models).

Our work takes a generative modelling approach. There is increasing interest in the use of generative approaches for machine learning, partly because they can be extended to work in a semi-supervised way. This enables unlabelled training data to contribute towards the model, potentially allowing more complex models to be learned from fewer labelled examples. Another motivation for generative modelling approaches is to enable missing data to be dealt with. Brain images – particularly hospital brain images – often have different fields of view from each other, with parts of the brain missing from some of the scans. Many machine learning approaches do not work well in the presence of missing data, so imputing missing information is an implicit part of the presented framework.

This work proposes a solution based on learning a form of shape and appearance model. The overall aim is to capture as much anatomical variability as possible using a relatively small number of latent variables. In addition to 3D brain image data, a number of other types of images will be used to illustrate other aspects of the very general framework that we present.

## 2 Methods

The proposed framework builds on many of the ideas presented in the principal geodesic analysis work of Zhang and Fletcher (2015). Modifications involve extending the framework to use a Gauss-Newton optimisation strategy, incorporating a variety of appearance noise models and also using a different overall form of regularisation. This section is divided into two main sections. The first of these describes the overall generative model, whereas the second describes the algorithm for fitting the model. Some of the notation used in this section is explained in Appendix A.

### 2.1 Generative model

The basic idea is that both shape and appearance may be modelled by linear combinations of spatial basis functions, and the objective is to automatically learn the best set of basis functions and latent variables from some collection of images. This is essentially a form of factorisation of the data. Each of the *N* images will be denoted by $f_{n}\in R^{M},$ where *M* is the number of pixels/voxels in an image, 1 ≤ *n* ≤ *N*, and the entire collection of images by **F**. An *appearance model* for the *n*th image is constructed from a linear combination of basis functions, such that

(2) $$a_{n}=\mu +W^{a}z_{n}.$$

Here, **W**^*a*^ is a matrix containing *K* columns of appearance basis functions, and **z**_*n*_ is a vector of *K* latent variables for the *n*th image. The vector ***μ*** is a mean image, with the same dimensions as a column of **W**^*a*^.

The *shape model* (used by Zhang and Fletcher (2015)) is encoded similarly, where initial velocity fields are computed by

(3) $$v_{n}=W^{v}z_{n}.$$

The Large-Deformation Diffeomorphic Metric Mapping (LDDMM) framework (Beg et al., 2005) is used, which allows images to be warped by smooth, invertible one-to-one mappings. Diffeomorphic deformations (*ψ_n_*) are computed from each **v**_*n*_ by a procedure known as “geodesic shooting”, which is presented in Algorithm 4 of Section 2.2.3.

From a probabilistic perspective, the likelihood can be summarised by

(4) $$p\left(f_{n}|z_{n},\mu ,W^{a},W^{v}\right)=p\left(f_{n}|a_{n}\left(\psi_{n}\right)\right),$$

where **a**(*ψ*) denotes warping the entire **a** by the deformation *ψ*. Different forms of noise model are presented in Section 2.1.2, but for convenience, we use the generic definition

(5) $$J\left(f_{n},z_{n},\mu ,W^{a},W^{v}\right)=-\ln p\left(f_{n}|z_{n},\mu ,W^{a},W^{v}\right).$$

In practice, a small amount of regularisation is imposed on the mean (***μ***) by assuming it is drawn from a multivariate Gaussian distribution of precision **L**^*μ*^ (see Section 2.1.3)

(6) $$p\left(\mu \right)=N\left(\mu |0,{\left(L^{\mu }\right)}^{-1}\right).$$

A weighted sum of two strategies for regularising estimates of the basis functions (**W**^*a*^ and **W**^*v*^) and latent variables (**z**_*n*_) is used, which are:

1. The first strategy involves separate priors on the basis functions, and on the latent variables. Each of the basis functions is assumed to be drawn from zero-mean highly multivariate Gaussian, parameterised by very large and sparse precision matrices. Possible forms of the matrices for regularising shape (**L**^*v*^) are described in Section 2.1.1, whereas those for appearance (**L**^*a*^) are described in Section 2.1.3. Priors for the basis functions (see Discussion section regarding scaling by *N*) are
  (7) $$p(W^{v})=\prod_{k=1}^{K}N\left(w_{k}^{v}|0,{\left(NL^{v}\right)}^{-1}\right),$$
  (8) $$p(W^{a})=\prod_{k=1}^{K}N\left(w_{k}^{a}|0,{\left(NL^{a}\right)}^{-1}\right).$$
  The latent variables (**Z**) are assumed to be drawn from zero-mean multivariate Gaussian distributions, parameterised by a precision matrix (**A**) that is derived from the data.^1^
  (9) $$p\left(z_{n}|A\right)=N\left(z_{n}|0,A^{-1}\right).$$
  The model assumes that matrix **A** is drawn from a Wishart distribution.
  (10) $$p(A)=W_{K}\left(A|\Lambda_{0},\nu_{0}\right)=\frac{{\left|A\right|}^{(\nu_{0}-K-1)/2}\exp\left(-\frac{1}{2}Tr\left(\Lambda_{0}^{-1}A\right)\right)}{2^{(\nu_{0}\;K)/2}{\left|\Lambda_{0}\right|}^{\nu_{0}/2}\Gamma_{K}\left(\frac{\nu_{0}}{2}\right)},$$
  where Γ_*K*_ is the multivariate gamma function. This prior can be made as uninformative as possible by using $\nu_{0}=K$ and $\Lambda_{0}=I/\nu_{0},$ where **I** is an identity matrix. In general, **Λ**_0_ should be a positive definite symmetric matrix, with *ν*_0_ ≥ *K* so that the distribution can be normalised.
2. The second strategy (used by Zhang and Fletcher (2015)) is a pragmatic solution to ensuring that enough regularisation is used.
  (11) $$\ln p(Z,W^{a},W^{v})=-\frac{1}{2}Tr(ZZ^{T}({\left(W^{a}\right)}^{T}L^{a}W^{a}+{\left(W^{v}\right)}^{T}L^{v}W^{v}))+\text{const}$$
  This strategy imposes smoothness on the reconstructions by assuming penalties based on $\ln N(W^{a}z_{n}|0,L^{a})$ and $\ln N(W^{v}z_{n}|0,L^{v}),$ in a similar way to more conventional regularisation approaches.

**Fig. 1:**
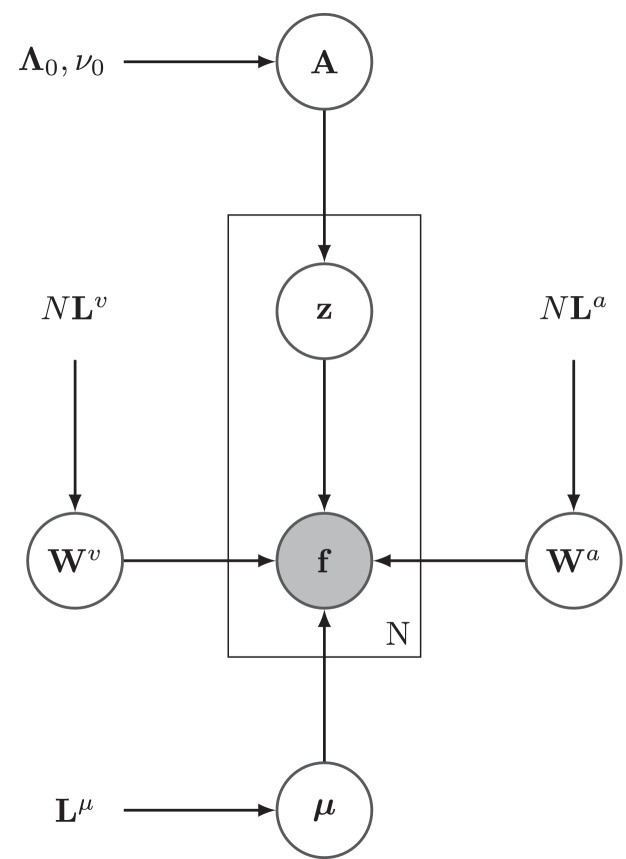
A graphical representation of the model (showing only the 1st strategy). Gray circles indicate observed data, whereas white circles indicate variables that are either estimated (**W**^*v*^, **W**^*a*^, ***μ*** and **z**) or marginalised out (**A**). The plate indicates replication over all images.

The weighting of the two strategies is controlled by user-specified weights *λ*_1_ and *λ*_2_. When everything is combined (see Fig. 1), the following joint log-probability is obtained

(12) $$\ln p(F,\mu ,W^{a},W^{v},A,Z)=-\sum_{n=1}^{N}J\left(f_{n},z_{n},\mu ,W^{a},W^{v}\right)-\frac{1}{2}\mu^{T}L^{\mu }\mu -\frac{\lambda_{1}N}{2}\left(Tr\left({\left(W^{a}\right)}^{T}L^{a}W^{a}\right)+Tr\left({\left(W^{v}\right)}^{T}L^{v}W^{v}\right)\right)+\frac{\lambda_{1}}{2}\left(\left(N+\nu_{0}-K-1\right)\ln\left|A\right|-Tr\left(\left(ZZ^{T}+\Lambda_{0}^{-1}\right)A\right)\right)-\frac{\lambda_{2}}{2}Tr\left(ZZ^{T}\left({\left(W^{a}\right)}^{T}L^{a}W^{a}+{\left(W^{v}\right)}^{T}L^{v}W^{v}\right)\right)+\text{const}.$$

The model fitting procedure is described in Section 2.2. Ideally, the procedure would compute distributions for all variables, such that uncertainty was dealt with optimally. Unfortunately, this is computationally impractical for the size of the datasets involved. Instead, only point estimates are made for the latent variables (${\hat{z}}_{n}$) and various parameters ($\hat{\mu },$ **W**^*a*^, **W**^*v*^), apart from **A**, which is inferred within a variational Bayesian framework.

The approach also allows an alternative formulation, whereby shapes and appearances are modelled separately by having some of the latent variables control appearance, and others control shape. This may be denoted by

(13) $$a_{n}=\mu +\sum_{k=1}^{K^{a}}w_{k}^{a}z_{kn},$$

(14) $$v_{n}=\sum_{k=1}^{K^{v}}w_{k}^{v}z_{mn}\text{,}\;\text{where}\;m=K^{a}+k.$$

For simplicity, only the form where each latent variable controls both shape and appearance is described in detail. This is the form used in active appearance models (Cootes et al., 2001). Note however, that in the form where shape and appearance are controlled by separate latent variables, the precision matrix **A** still encodes covariance between the two types of variables. This means that latent variables controlling either shape or appearance are not estimated completely independently.

#### 2.1.1 Differential operator for shape model

The precision matrix used in (Eq. (7)) has the form

(15) $$v^{T}L^{v}v=\int_{x\in \Omega }\left(\omega_{0}^{v}{∥v\left(x\right)∥}^{2}+\omega_{1}^{v}{∥\nabla v\left(x\right)∥}^{2}+\omega_{2}^{v}{∥\nabla^{2}v\left(x\right)∥}^{2}\right)dx+\;\int_{x\in \Omega }\left(\frac{\omega_{3}^{v}}{4}{∥Dv\left(x\right)+{\left(Dv\left(x\right)\right)}^{T}∥}_{F}^{2}+\omega_{4}^{v}Tr{\left(Dv\left(x\right)\right)}^{2}\right)dx$$

where ‖ · ‖_*F*_ denotes the Frobenius norm (the square root of the sum of squares of the matrix elements) and *D* denotes the operator computing Jacobian tensors. The above integral is defined in Sobolev space, which is a weighted Hilbert space where spatial derivatives, up to a certain degree, are accounted for. Five user-specified hyper-parameters are involved:

• $\omega_{0}^{v}$ controls absolute displacements, and is typically set to be a very small value.
$\omega_{1}^{v}$ controls stretching, shearing and rotation.
$\omega_{2}^{v}$ controls bending energy. This ensures that the resulting velocity fields have smooth spatial derivatives.
$\omega_{3}^{v}$ controls stretching and shearing (but not rotation).
$\omega_{4}^{v}$ controls the divergence, which in turn determines the amount of volumetric expansion and contraction.

Most of the regularisation in this work was based on a combination of the linear-elasticity (using *Lamé’s constants* $\omega_{3}^{v}$ and $\omega_{4}^{v}$) and bending energy ($\omega_{2}^{v}$) penalties. The effects of different forms of regularisation used for registration are illustrated in Ashburner and Ridgway (2012).

#### 2.1.2 Noise models

A number of different choices for the noise model are available for (Eq. (4)), each suitable for modelling different types of image data. These models are based on $p(f_{n}|a_{n}^{\prime }),$ which leads to an “energy” term (*J*) that drives the model fitting and is assumed to be independent across voxels

(16) $$a_{n}^{\prime }=\Psi_{n}\left(\mu +W^{a}z_{n}\right)$$

(17) $$J(a_{n}^{\prime })=-\ln p\left(f_{n}|a_{n}^{\prime }\right)=-\sum_{m=1}^{M}\ln p\left(f_{mn}|a_{mn}^{\prime }\right).$$

Because the approach is generative, missing data are handled by simply ignoring those voxels where there is no information. By doing this, they do not contribute towards the objective function and play no role in driving the model fitting. A number of different energy functions have been implemented for modelling different types of data. These are listed next.

##### 2.1.2.1 Gaussian noise model

Mean-squares difference is a widely used objective functions for image matching, which is based on the assumption of stationary Gaussian noise. For an image consisting of *M* pixels or voxels, the function would be

(18) $$-J_{L_{2}}\left(a^{\prime }\right)=\ln p\left(f|a^{\prime },\sigma^{2}\right)=-\frac{M}{2}\ln\left(2\pi \right)-\frac{M}{2}\ln\sigma^{2}-\frac{1}{2\sigma^{2}}{∥f-a^{\prime }∥}_{2}^{2},$$

where || · ||_2_ denotes the Euclidean norm. The simplest approach to compute *σ*^2^ is to make a maximum likelihood estimate from the variance by

(19) $$\hat{\sigma^{2}}=\frac{1}{MN}\sum_{n=1}^{N}{∥f_{n}-a_{n}^{\prime }∥}_{2}^{2}.$$

##### 2.1.2.2 Logistic function with Bernoulli noise model

When working with binary images, such as single tissue type maps having voxels of zeros and ones (or values very close to zero or one), it may be better to work under the assumption that voxels are drawn from a Bernoulli distribution, which is a special case of the binomial distribution. For a single voxel,

(20) $$P\left(f|s\right)=s^{f}{\left(1-s\right)}^{1-f}.$$

The range 0 < *s* < 1 must be satisfied, which is achieved using a logistic sigmoid function

(21) $$s\left(a^{\prime }\right)=\frac{1}{1+\exp(-a^{\prime })}.$$

Putting these together leads to the matching function

(22) $$-J_{Bern}\left(a^{\prime }\right)=\ln P\left(f|a^{\prime }\right)=\sum_{m=1}^{M}\left(f_{m}a_{m}^{\prime }+\ln s\left(-a_{m}^{\prime }\right)\right).$$

##### 2.1.2.3 Softmax function with categorical noise model

If there are several binary maps to align simultaneously, for example maps of grey matter, white matter and background, then a categorical noise model is appropriate. A categorical distribution is a generalisation of the Bernoulli distribution, and also a special case of the multinomial distribution. The probability of a vector **f** of length *C*, such that *f_c_* ∈ {0, 1} and $\sum_{c=1}^{C}f_{c}=1,$ is given by

(23) $$P\left(f|s\right)=\prod_{c=1}^{C}s_{c}^{f_{c}},$$

where *s_c_* > 0 and $\sum_{c=1}^{C}s_{c}=1$. The constraint on **s** is enforced by using a softmax function.

(24) $$s_{c}\left(a^{\prime }\right)=\frac{\exp a_{c}^{\prime }}{\sum_{c=1}^{C}\exp a_{c}^{\prime }}$$

Using the “log-sum-exp trick”, numerical overflow or underflow can be prevented by first subtracting the maximum of **a**, so

(25) $$s_{c}\left(a^{\prime }\right)=\frac{\exp(a_{c}^{\prime }-a^{*})}{\sum_{c=1}^{C}\exp\left(a_{c}^{\prime }-a^{*}\right)}\text{,}\;\text{where}\;a^{*}=\max\left\{a_{1}^{\prime },\ldots ,a_{C}^{\prime }\right\}$$

Noting that each image is now a matrix of *M* voxels and *C* classes, the objective function can then be computed as

(26) $$-J_{cat}\left(A^{\prime }\right)=\ln P(F|A^{\prime })=\sum_{m=1}^{M}\left(\sum_{c=1}^{C}a_{mc}^{\prime }f_{mc}-a^{*}-\log\left(\sum_{c=1}^{C}\exp\left(a_{mc}^{\prime }-a_{m}^{*}\right)\right)\right)$$

#### 2.1.3 Differential operator for appearance model

Regularisation is required for the appearance variability, as it helps to prevent the appearance model from absorbing too much of the variance, at the expense of the shape model. This differential operator (again based on a Sobolev space) is used in Eqs. (6) and (8), and controlled by three hyper-parameters.

(27) $$a^{T}L^{a}a=\int_{x\in \Omega }\left(\omega_{0}^{a}{∥a\left(x\right)∥}^{2}+\omega_{1}^{a}{∥\nabla a\left(x\right)∥}^{2}+\omega_{2}^{a}{∥\nabla^{2}a\left(x\right)∥}^{2}\right)dx$$

### 2.2 Algorithm for model fitting

A highly simplified version of what was implemented is shown in Algorithm 1. The model fitting approach involves alternating between computing the shape and appearance basis functions (plus a few other variables - *Step-1*), and re-estimating the latent variables (*Step-2*). For better convergence of the basis function updates, an orthogonalisation step is included in each iteration.

**Algorithm 1:**
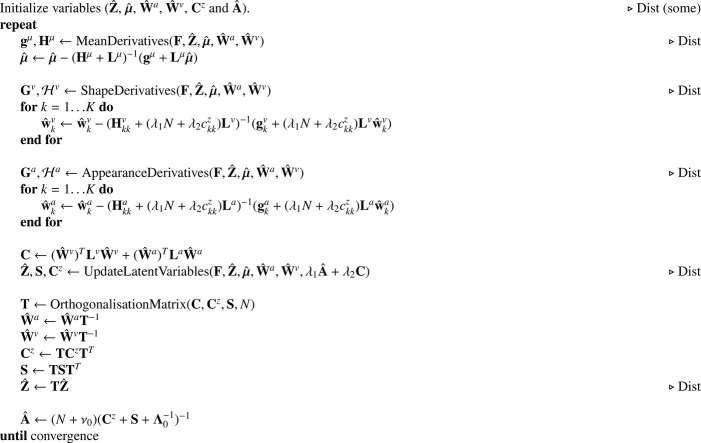
Shape and appearance model.

Step-1 relies on Gauss-Newton updates of three elements: the mean template (***μ***), shape subspace (**W**^*a*^) and appearance subspace (**W**^*v*^). These updates have the general form of $w\leftarrow w-{\left(H+L\right)}^{-1}\left(g+Lw\right),$ where **L** is a very sparse Toeplitz or circulant matrix encoding spatial regularisation, and **H** encodes a field of small matrices that are easy to invert. The full-multigrid method, described in Ashburner (2007), is particularly well suited to solving this type of problem.

Step-2 involves updating the latent variables (**Z**) and Gaussian prior (**A**). To break the initial symmetry, the latent variables are all initialised randomly, while ensuring that $\hat{Z}{\hat{Z}}^{T}=NI$. Correspondingly, matrix **C**^*z*^ is initialised to *N***I** and $\hat{A}$ is initialised to $\left(N+\nu_{0}\right){\left(NI+\Lambda_{0}^{-1}\right)}^{-1}$. An initial estimate for ***μ*** is computed from the unaligned data in a fairly straightforward way, whereas ${\hat{W}}^{a}$ and ${\hat{W}}^{v}$ are both initialised to zero.

Comments in Algorithm 1 saying “Dist” indicate which steps should be modified for running within a distributed privacy-preserving framework. The idea here is that the main procedure would be run on the “master” computer, whereas various functions would be run on the “worker” machines on which the data reside. These workers would only pass aggregate data back to the master, whereas the latent variables, which explicitly encode information about individuals, would remain on the workers. As the algorithm is described here, the images (**F**) and estimated latent variables $\hat{Z}$ are passed back and forth between the master and workers, but this need not be the case. If these data and variables were all to reside on the worker machines, the master machine would still be able to run using only the aggregate data.

For simplicity, Algorithm 1 does not include functions for computing variances (*σ*^2^ used by the Gaussian noise model), etc., and these variables are not shown to be passed to the various functions that use them. However, it should be easy to see how these changes would be incorporated in practice. Also, the illustration does not show any steps requiring the objective function, which include various backtracking line-searches to ensure that parameter updates cause the objective function to improve each time. In practice, the algorithm is run for a fixed number of iterations, although the log-likelihood could be used to determine when to stop.

#### 2.2.1 Updating the mean ($\hat{\mu }$)

From (Eq. (12)), we see that a point estimate of the mean (***μ***) may be computed by

(28) $$\hat{\mu }=\underset{\mu }{arg\;min}\left(\frac{1}{2}\mu^{T}L^{\mu }\mu +\sum_{n=1}^{N}J\left(f_{n},{\hat{z}}_{n},\mu ,{\hat{W}}^{a},{\hat{W}}^{v}\right)\right).$$

In practice, this log probability is not fully maximised with respect to ***μ*** at each iteration. Instead, $\hat{\mu }$ is updated by a single Gauss-Newton iteration. This requires gradients and Hessians computed as shown in Algorithm 2, which simply involves summing over those computed for the individual images. A small amount of regularisation is used for the estimate of the mean, which is important in situations where it can help to smooth over some of the effects of missing data.

**Algorithm 2:**
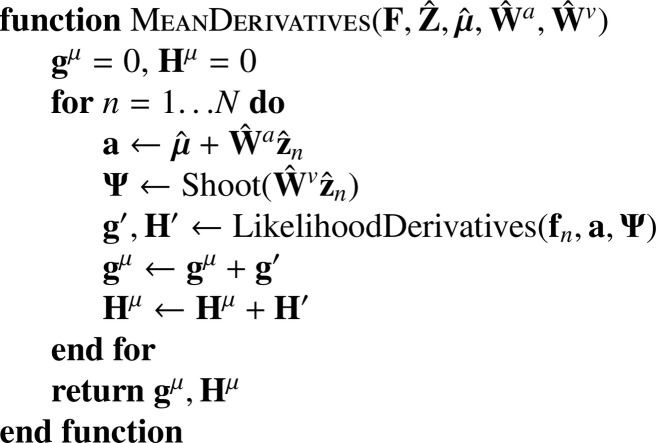
Computing gradients and Hessians for mean.

#### 2.2.2 Likelihood derivatives

The algorithm can be run using a number of different noise models, and the gradients and Hessians involved in the Gauss-Newton updates depend upon the one used.

##### 2.2.2.1 Gaussian model

Algorithm 3 shows derivatives for the Gaussian noise model (Eq. (18)). For a single voxel, this is based on

(29) $$\frac{dJ_{L_{2}}}{da^{\prime }}=\frac{1}{\sigma^{2}}\left(a^{\prime }-f\right)\;\text{and}\;\frac{d^{2}J_{L_{2}}}{da^{\prime 2}}=\frac{1}{\sigma^{2}}$$

For voxels where data is missing, both $J_{L_{2}}$ and $\frac{dJ_{L_{2}}}{da^{\prime }}$ are assumed to be zero. Using matrix notation, the objective function for an image is therefore

(30) $$J^{\prime }=\frac{1}{2\sigma^{2}}{\left(\Psi a-f\right)}^{T}\left(\Psi a-f\right)+\frac{M}{2}\left(\ln\left(\sigma^{2}\right)+\ln\left(2\pi \right)\right).$$

The gradients and Hessians, with respect to variations in **a**, are

(31) $$g^{\prime }=\Psi^{T}\left(\frac{1}{\sigma^{2}}\left(\Psi a-f\right)\right)$$

(32) $$H^{\prime }=\frac{1}{\sigma^{2}}\Psi^{T}\Psi$$

In practice, the Hessian (**H**′) is approximated by a diagonal matrix

(33) $$H^{\prime }≃\text{diag}\left(\Psi^{T}1\frac{1}{\sigma^{2}}\right),$$

where **1** is a vector of ones. This approximation works in the optimisation because all rows of **Ψ** sum to 1, so for any vector **d** of the right dimension, the rows of **Ψ**^*T*^diag(**d**)**Ψ** sum to **Ψ**^*T*^**d**. Because (for trilinear interpolation) all elements of **Ψ** are greater than or equal to zero, so if all elements of **d** are non-negative, then all eigenvalues of $\text{diag}\left(\Psi^{T}d\right)-\Psi^{T}\text{diag}\left(d\right)\Psi$ are greater than or equal to zero.^2^These non-negative eigenvalues ensure that our approximation to the Hessian (Eq. (33)) is more positive semi-definite than (Eq. (32)).

**Algorithm 3:**
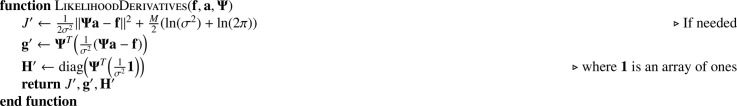
Likelihood derivatives for Gaussian noise model.

##### 2.2.2.2 Binary model

For the Bernoulli noise model with the sigmoidal squashing function (Eq. (22)), some modifications are made to the gradient and Hessian of Algorithm 3, based on the derivatives

(34) $$\frac{dJ_{Bern}}{da^{\prime }}=s\left(a^{\prime }\right)-f\;\text{and}\;\frac{d^{2}J_{Bern}}{da^{\prime 2}}=s\left(a^{\prime }\right)\left(1-s\left(a^{\prime }\right)\right).$$

Using matrix notation (where **s** ≡ *s*(**a**)), the gradients and Hessians are

(35) $$g^{\prime }=\Psi^{T}\left(\Psi s-f\right)$$

(36) $$H^{\prime }=\Psi^{T}\text{diag}\left(s\right)\text{diag}\left(1-s\right)\Psi ≃\text{diag}\left(\Psi^{T}\text{diag}\left(s\right)\left(1-s\right)\right)$$

##### 2.2.2.3 Categorical model

The categorical model with a softmax squashing function (Eq. (26)) would use the gradients and Hessians

(37) $$\frac{dJ_{cat}}{da_{k}^{\prime }}=s_{k}\left(a^{\prime }\right)-f_{k}\text{,}\;\text{where}\;s\left(a^{\prime }\right)=\frac{\exp a^{\prime }}{\sum_{k=1}^{K}\exp a_{k}^{\prime }}$$

(38) $$\frac{d^{2}J_{cat}}{da_{k}^{\prime }a_{l}^{\prime }}=s_{k}\left(a^{\prime }\right)\left(\delta_{jk}-s_{j}\left(a^{\prime }\right)\right),$$

where *δ_jk_* is the Kronecker delta function. Computation of the gradients and the approximation of the Hessian follow similar lines to those for the binary and Gaussian models.

#### 2.2.3 Geodesic shooting

Algorithm 4 shows how diffeomorphic deformations are computed from the initial velocities via a Geodesic shooting procedure. In the presented algorithm, *Dψ* denotes the Jacobian tensor field of *ψ*, and (*Dψ*)^*T*^*u* indicates a pointwise multiplication with the transpose of the Jacobian. |*Dψ*| denotes the field of Jacobian determinants. *Lv* in the continuous framework is equivalent to the matrix multiplication **L**^*v*^**v** in the discrete framework. The operation *L^g^u* denotes applying the inverse of *L* to *u*, such that $LL^{g}u=u$. In practice, this is a deconvolution, which is computed using fast Fourier transform (FFT) methods to obtain the Green’s function (Bro-Nielsen and Gramkow, 1996). Because of this, the boundary conditions for the velocity fields (and other spatial basis functions) are assumed to be periodic. Much has already been written about the geodesic shooting procedure, so the reader is referred to Miller et al. (2006) and Ashburner and Friston (2011) for further information.

**Algorithm 4:**
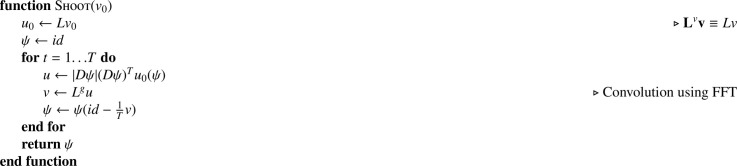
Geodesic shooting via Euler integration.

#### 2.2.4 Updating appearance basis functions (${\hat{W}}^{a}$)

Appearance basis functions are optimised by

(39) $${\hat{W}}^{a}=\underset{W^{a}}{\arg\min}(\frac{1}{2}Tr\left(\left(\lambda_{1}NI+\lambda_{2}\hat{Z}{\hat{Z}}^{T}\right){\left(W^{a}\right)}^{T}L^{a}W^{a}\right)+\sum_{n=1}^{N}J\left(f_{n},{\hat{z}}_{n},\hat{\mu },W^{a},{\hat{W}}^{v}\right)).$$

The first step involves computing the gradients and Hessians, which is shown in Algorithm 5. Note that this only shows the computation of gradients and Hessians for the Gaussian noise model, and that slight modifications are required when using other forms of noise model. Gradients and Hessians for updating these basis functions (**W**^*a*^) are similar to those for the mean updates, except for weighting based on the current estimates of the latent variables. Note that for this approach to work effectively, the rows of $\hat{Z}$ should be orthogonal to each other, which is explained further in Section 2.2.8. Note that only a single Gauss-Newton step is performed in each iteration, so the objective function in (Eq. (39)) is not fully optimised, but merely improved over its previous value.

**Algorithm 5:**
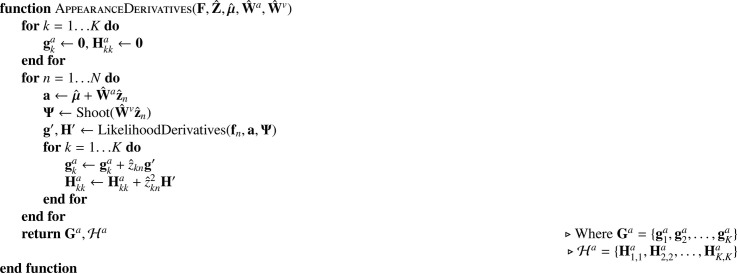
Computing gradients and Hessians for appearance.

#### 2.2.5 Updating shape basis functions (${\hat{W}}^{v}$)

Shape basis functions are optimised by

(40) $$\begin{array}{c} {\hat{W}}^{v}=\underset{W^{v}}{\arg\min}(\frac{1}{2}Tr\left(\left(\lambda_{1}NI+\lambda_{2}\hat{Z}{\hat{Z}}^{T}\right){\left(W^{v}\right)}^{T}L^{v}W^{v}\right)\\ +\sum_{n=1}^{N}J\left(f_{n},{\hat{z}}_{n},\hat{\mu },{\hat{W}}^{a},W^{v}\right)). \end{array}$$

A single Gauss-Newton iteration is used to update the basis functions of the shape model (**W**^*v*^), which is done in such a way that changes to **W**^*v*^ improve the objective function with respect to its previous value, rather than fully optimise . (Eq. (40)). While most Gauss-Newton iterations improve the fit, a backtracking line search is included to ensure that they do not overshoot. As for updating **W**^*a*^, this requires the rows of $\hat{Z}$ to be orthogonal to each other. The strategy for computing gradients and Hessians is shown in Algorithm 6.

**Algorithm 6:**
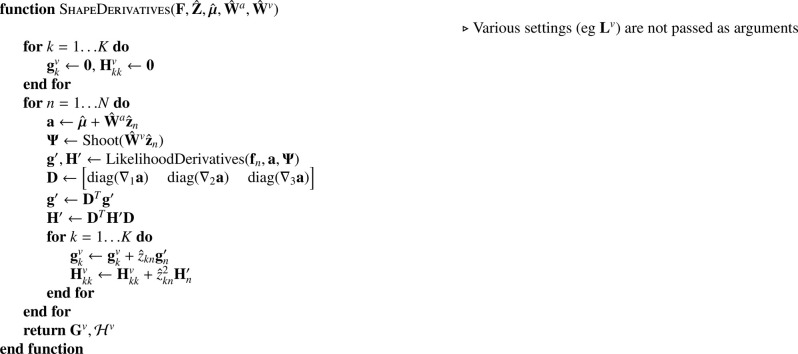
Computing gradients and Hessians for shape.

#### 2.2.6 Updating latent variables (${\hat{z}}_{n}$)

The modes of the latent variables are updated via a Gauss-Newton scheme (shown in Algorithm 7), similar to that used by Friston et al. (1995),Cootes et al. (2001) and Cootes and Taylor (2001).

(41) $${\hat{z}}_{n}=\underset{z_{n}}{\arg\min}\left(J(f_{n},z_{n},\mu ,{\hat{W}}^{a},{\hat{W}}^{v}\right)+\frac{1}{2}z_{n}^{T}\left(\lambda_{1}\hat{A}+\lambda_{2}{\left({\hat{W}}^{a}\right)}^{T}L^{a}{\hat{W}}^{a}+\lambda_{2}{\left({\hat{W}}^{v}\right)}^{T}L^{v}{\hat{W}}^{v}\right)z_{n})$$

**Algorithm 7:**
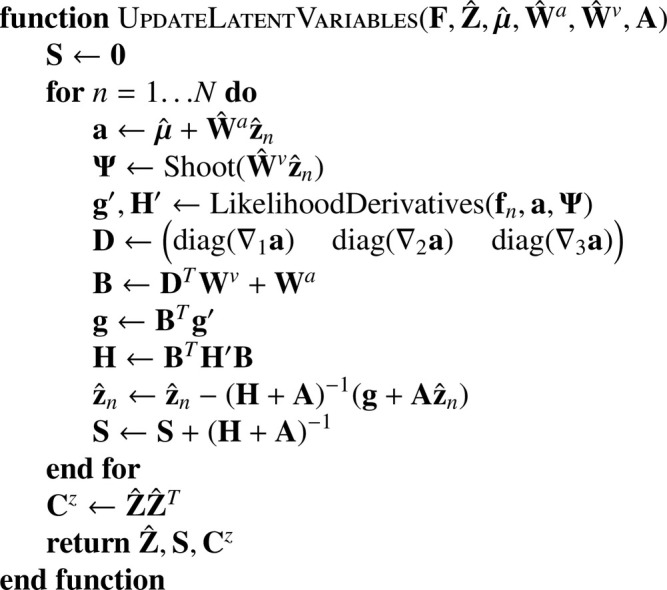
Updating latent variables.

The inverse of the (approximate) Hessians allows a Gaussian approximation of the uncertainty with which the latent variables are updated to be computed (“Laplace approximation”). This is the **S** matrix, which is combined with $\hat{Z}{\hat{Z}}^{T}$ (returned as **C**^*z*^) and used to re-compute $\hat{A}$.

#### 2.2.7 Expectation of the precision matrix ($\hat{A}$)

This work uses a variational Bayesian approach for approximating the distribution of **A**, which is a method described in more detail by textbooks, such as Bishop et al. (2006) or Murphy (2012). Briefly, it involves taking the joint probability of (Eq. (12)), discarding terms that do not involve **A**, and substituting the expectations of the other parameters into the expression. This leads to the following approximating distribution, which can be recognised as Wishart.

(42) $$\ln q(A)=\frac{1}{2}\left(N+\nu_{0}-K-1\right)\ln\det\left|A\right|-\;\frac{1}{2}Tr\left((E\left[ZZ^{T}\right]+\Lambda_{0}^{-1})A\right)+\text{const}=\ln W_{K}\left(A|\Lambda ,\nu \right),$$

where $\Lambda ={\left(E\left[ZZ^{T}\right]+\Lambda_{0}^{-1}\right)}^{-1}$ and $\nu =\nu_{0}+N$. In practice, $E[ZZ^{T}]$ is approximated by $C^{z}+S,$ described previously. Other steps in the algorithm use the expectation of **A**, which (see Appendix B of Bishop et al. (2006)) is

(43) $$\hat{A}=E\left[A\right]=\nu \Lambda .$$

#### 2.2.8 Orthogonalisation

The strategy for updating ${\hat{W}}^{a}$ and ${\hat{W}}^{v}$ involves some approximations, which are needed in order to save memory and computation. This approximation is related to the Jacobi iterative method for determining the solutions to linear equations, which is only guaranteed to converge for diagonally dominant matrices. Rather than work with the Hessian for the entire **W** matrix together, only the Hessians for each column of **W** are computed by Algorithms 5 and 6. This corresponds with a block diagonal Hessian matrix for the entire **W**, which has the form

(44) $$H=\left(\begin{array}{cccc} H_{11} & 0 & \cdots & 0\\ 0 & H_{22} & \cdots & 0\\ \vdots & \vdots & \ddots & \vdots \\ 0 & 0 & \cdots & H_{KK} \end{array}\right).$$

More stable convergence can be achieved by transforming the basis functions and latent variables in order to minimise the amount of signal that would be in the off-diagonal blocks, thus increasing the diagonal dominance of the system of equations. In situations where diagonal dominance is violated, convergence can still be achieved by decreasing the update step size. This is analogous to using a weighted Jacobi iteration, where in practice the weights are found using a backtracking line-search.

Signal in the off-diagonal blocks is reduced by orthogonalising the rows of $\hat{Z}$. This is achieved by finding a transformation, **T**, such that $T\hat{Z}{\left(T\hat{Z}\right)}^{T}$ and ${\left({\hat{W}}^{v}T^{-1}\right)}^{T}L^{v}{\hat{W}}^{v}T^{-1}$ + ${\left({\hat{W}}^{a}T^{-1}\right)}^{T}L^{a}{\hat{W}}^{a}T^{-1}$ are both diagonal matrices. Transformation **T** is derived from an eigendecomposition of the sufficient statistics, whereby the symmetric positive definite matrices are decomposed into diagonal (**D**^*z*^ and **D**^*w*^) and orthonormal (**V**^*z*^ and **V**^*w*^) matrices, such that

(45) $$V^{z}D^{z}{\left(V^{z}\right)}^{T}=C^{z},$$

(46) $$V^{w}D^{w}{\left(V^{w}\right)}^{T}=C,$$

where $C^{z}=\hat{Z}{\hat{Z}}^{T}$ and $C={\left({\hat{W}}^{v}\right)}^{T}L^{v}{\hat{W}}^{v}+{\left({\hat{W}}^{a}\right)}^{T}L^{a}{\hat{W}}^{a}$.

A further singular value decomposition is then used, giving

(47) $$UDV^{T}={\left(D^{w}\right)}^{\frac{1}{2}}{\left(V^{w}\right)}^{T}V^{z}{\left(D^{z}\right)}^{\frac{1}{2}}.$$

The combination of various matrices is used to give an initial estimate of the transform

(48) $$T=DV^{T}{\left(D^{z}\right)}^{-\frac{1}{2}}{\left(V^{z}\right)}^{T}.$$

The above **T** matrix could be used to render the matrices orthogonal, but their relative scalings would not be optimal. The remainder of the orthogonalisation procedure involves an iterative strategy similar to expectation maximisation, where the aim is to estimate some diagonal scaling matrix **Q** with which to multiply **T**. This matrix is parameterised by a set of parameters **q**, such that

(49) Q=diag(expq).

The first step of the iterative scheme involves re-computing $\hat{A},$ as described in Section 2.2.7, but incorporating the current estimates of **QT**.

(50) $$\hat{A}=\nu \Lambda =\left(N+\nu_{0}\right){\left(QT\left(C^{z}+S\right){\left(QT\right)}^{T}+\Lambda_{0}^{-1}\right)}^{-1}.$$

The next step in the iterative scheme is to re-estimate **q**, such that

(51) $$\hat{q}=\underset{q}{\arg\min}(Tr\left(\text{diag}\left(\exp\left(-q\right)\right){\left(T^{-1}\right)}^{T}CT^{-1}\text{diag}\left(\exp\left(-q\right)\right)\right)+Tr\left(\text{diag}\left(\exp q\right)TC^{z}T^{T}\text{diag}\left(\exp q\right)\hat{A}\right)).$$

This is achieved via a Gauss-Newton update, which uses first and second derivatives with respect to **q**. The overall strategy is illustrated in Algorithm 8, which empirically is found to converge well.

**Algorithm 8:**
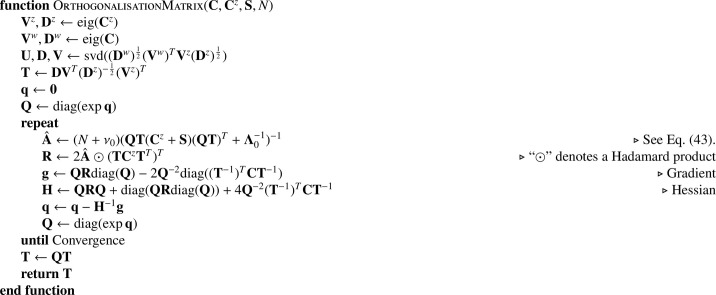
Orthogonalising the variables.

## 3 Results

To show the general applicability of the approach, evaluations were performed with a number of datasets of varying characteristics. Our implementation^3^ is written in a mixture of MATLAB and C code (MATLAB “mex” files for the computationally expensive parts).

### 3.1 Qualitative 2D experiments with faces

After years of exposure to faces, most people can identify whether an image of a face is plausible or not, so images of human faces provide a good qualitative test of how well the algorithm can model biological variability.

The straight on views from the Karolinska Directed Emotional Faces (KDEF) data-set (Lundqvist et al., 1998) were used to make a visual assessment of how well the algorithm performs. This data-set consisted of photographs of 70 participants, holding seven different facial expressions, which was repeated twice. Some of the images were excluded because they were systematically brighter (47 images) or had different dimensions (one image), leaving a final dataset consisting of 932 colour images, which were downsampled to a size of 282 × 382. The original intensities were in the range of 0 to 255, but these values were re-scaled by 1/255.

**Fig. 2:**
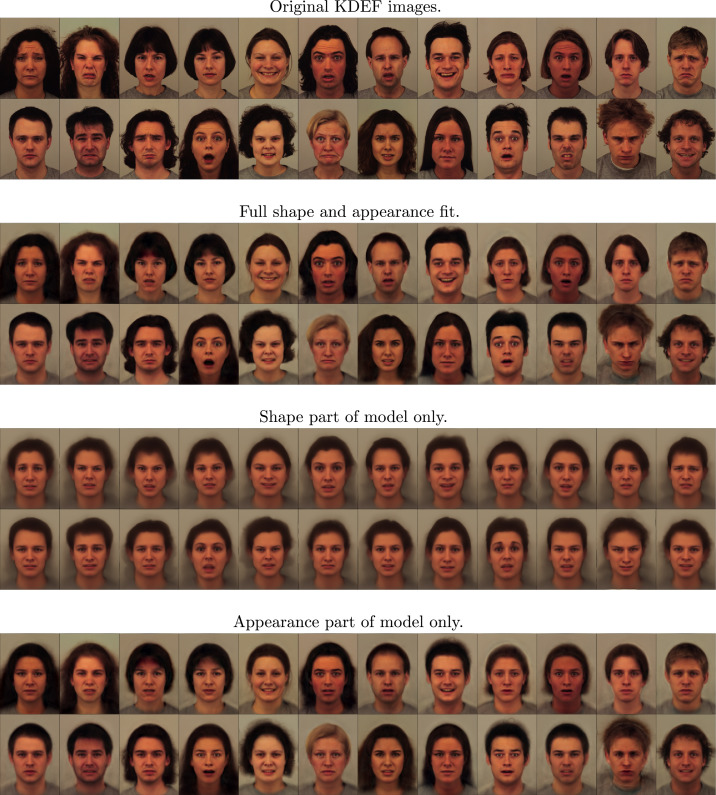
Shape and appearance fit shown for a randomly selected sample of the KDEF face images. (For interpretation of the references to colour in this figure legend, the reader is referred to the web version of this article.)

**Fig. 3:**
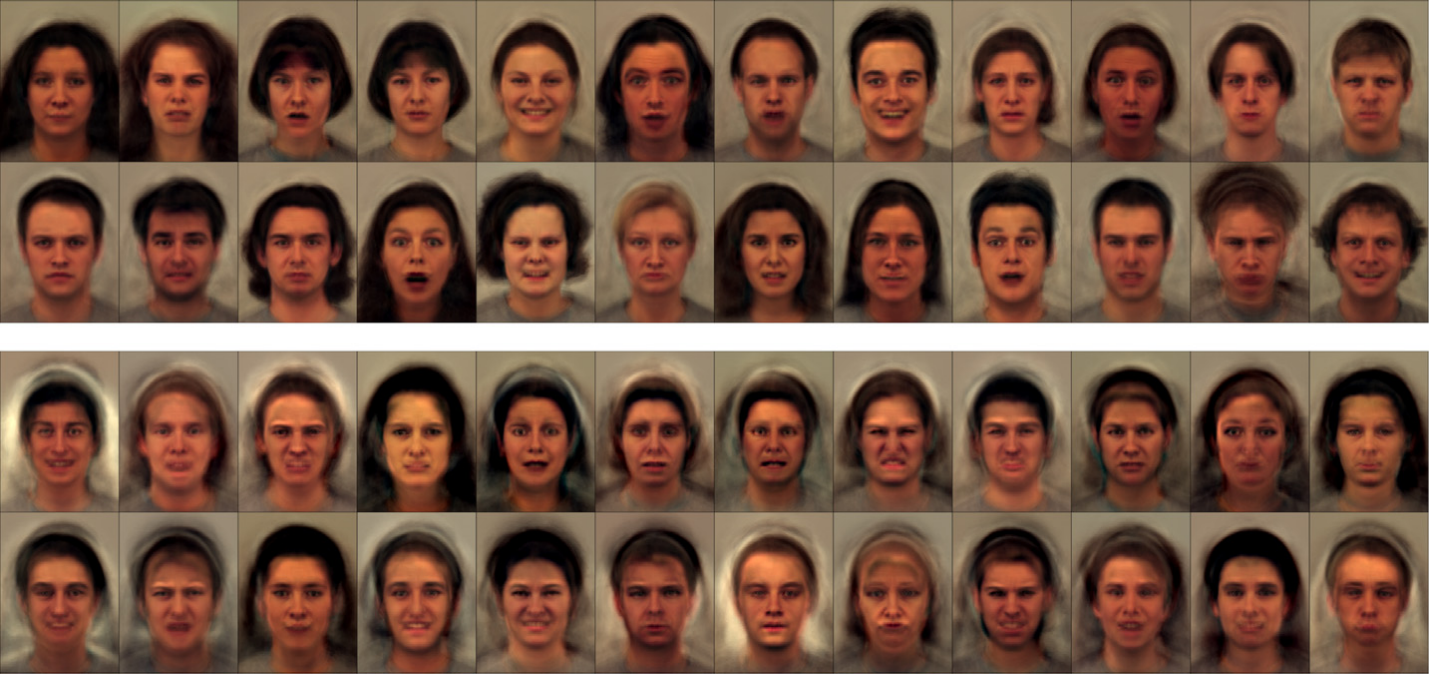
Fits using a simple 64-mode principal component analysis model are shown above (cf. Fig. 2), and random faces generated from the same PCA model are shown below (cf. Fig. 4). (For interpretation of the references to colour in this figure legend, the reader is referred to the web version of this article.)

A 64 eigenmode model was used (K=64), which assumed Gaussian noise. Model fitting (i.e., learning the shape and appearance basis functions, etc.) was run for 20 iterations, with $\nu_{0}=1000,$ λ=[15.20.8], $\omega^{a}=\left[4\;512\;64\right],$ $\omega^{\mu }=N\left[10^{-4}\;0.1\;0.1\right]$ and $\omega^{v}=\left[10^{-3}\;0\;16\;1\;1\right]$. It was fit to the entire field of view of the images, rather than focusing only on the faces, and some of the resulting fits are shown in Fig. 2. The first set of images are a random selection of the original data, with the full shape and appearance model fits shown immediately below. As can be seen, the fit is reasonably good - especially given that only 64 modes of variability were used, and that these have to account for a lot of variability of hair etc. Below these are the shape model fits, generated by warping the mean according to the estimated deformations (***μ***(*ψ_n_*)). The appearance fits are shown at the bottom (**a**_*n*_ from (Eq. (4))). Ideally, these reconstructions of appearance should be in perfect alignment with each other, which is not quite achieved in certain parts of the images. In particular, the thickness of the neck varies according to whether or not the people in the images have short or long hair. When looked at separately, the shape and appearance parts of the model do not behave quite so well, but when combined, they give quite a good fit. Fig. 3 shows a simple 64-mode principal component analysis (PCA) fit to the same data, which clearly does not capture variability quite as well as the shape and appearance model.

**Fig. 4:**
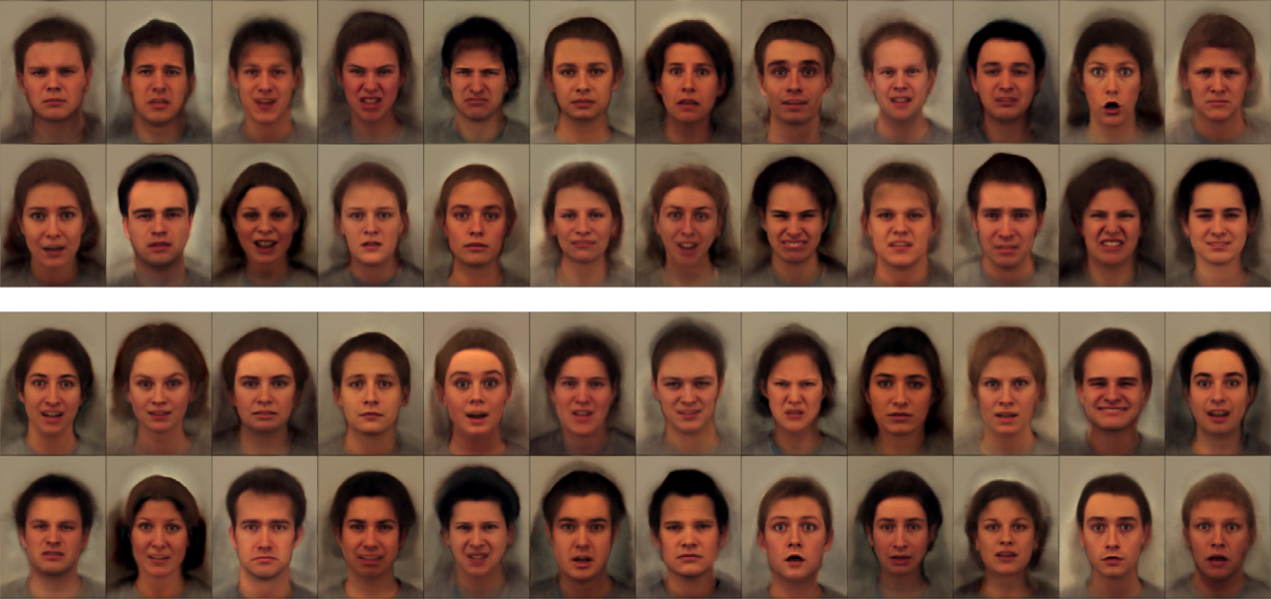
Random faces generated from the shape and appearance model. The lower set of faces were generated with the same latent variables as those shown in the upper set, except the values were multiplied by −1 and thus show a sort of “opposite” face. For example, if a face in the top set has a wide open mouth, then the mouth should be tightly closed in the corresponding image of the bottom set. (For interpretation of the references to colour in this figure legend, the reader is referred to the web version of this article.)

For these examples, there should really have been a distinction between inter-subject variability and intra-subject variability, using some form of hierarchical model for the latent variables. This type of hierarchical mixed-effects model is widely used for analysing multi-subject data within the neuroimaging field (Friston et al., 2002), and a number of works have applied mixed effects modeling to image registration (Datar, Muralidharan, Kumar, Gouttard, Piven, Gerig, Whitaker, Fletcher, 2012, Allassonnière, Durrleman, Kuhn, 2015).

#### 3.1.1 Simulating faces

Once the model is learned, it becomes possible to generate random faces from the estimated distribution. This involves drawing a random vector of latent variables $z∼N(0,{\hat{A}}^{-1}),$ and using these to reconstruct a face. Fig. 4 shows two sets of randomly generated faces, where the lower set used the same latent variables as the upper set, except that they were multiplied by −1. Although some of the random faces are not entirely plausible, they are much more realistic than faces generated from a simple 64-mode PCA model (shown in Fig. 3).

#### 3.1.2 Vector arithmetic

**Fig. 5:**
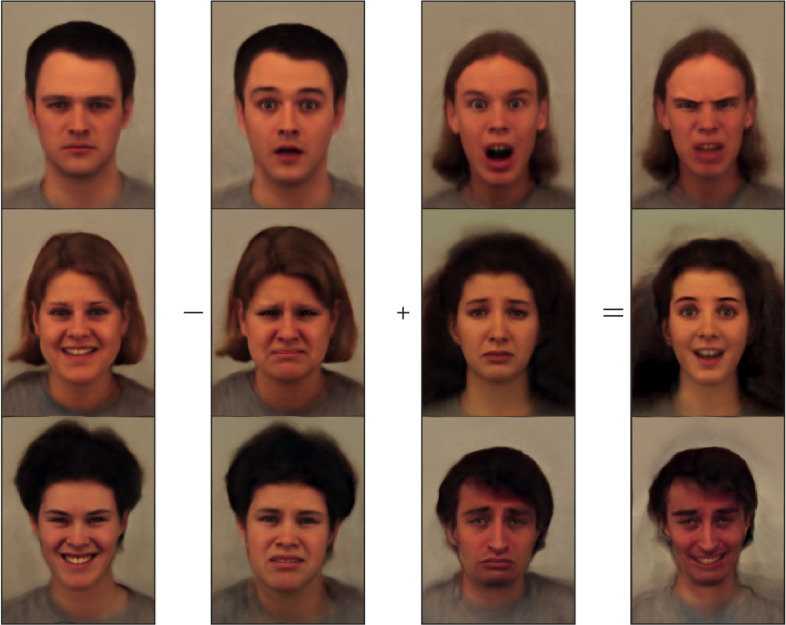
An example of simple linear additions and subtractions applied to the latent variables. The first three columns show the full shape and appearance model fits to various faces. Images in the right hand column were generated by making linear combinations of the latent variables that encode the images in the first three columns, and then reconstructing from these linear combinations. (For interpretation of the references to colour in this figure legend, the reader is referred to the web version of this article.)

In many machine learning applications, it is useful to be able to model certain non-linearities in the data in an approximately linear way, allowing more interpretable linear methods to be used while still achieving a good fit. Following Radford et al. (2015), this section shows that simple arithmetic on the latent variables can give intuitive results. The first three columns of Fig. 5 show the full shape and appearance model fits to various faces. Images in the right hand column of Fig. 5 were generated by making linear combinations of the latent variables that encode the images in the first three columns, and then reconstructing from these. Unlike arithmetic computed in pixel space (not shown), performing arithmetic on the vectors encoding the images gives reasonably plausible results.

### 3.2 2D experiments with MNIST

In this section, the behaviour of the approach using “big data” is assessed, which gives more of an idea of how this type of method may behave with some of the very large image datasets currently being collected. Instead of testing on a large collection of medical images, the approach was applied to a large set of tiny images of hand-written digits. MNIST^4^ (LeCun et al., 1998) is a modified version of the handwritten digits from the National Institute of Standards and Technology (NIST) Special Database 19. The dataset consists of a training set of 60,000 28 × 28 pixel images of the digits 0 to 9, along with a testing set of 10,000 digits. MNIST has been widely used for assessing the accuracy of machine learning approaches, and is used here as it allows behaviour of the current approach to be compared against the state-of-the-art pattern recognition methods.

In recent years, the medical imaging community has seen many of the established “old-school” approaches replaced by deep learning, but in doing so, “have we thrown the baby out with the bath water?”.^5^ There may still be widely used concepts from orthodox medical imaging (i.e., not deep learning) that are still useful. In particular, geometric transformations of images are now finding their way into various machine learning approaches (e.g. Hinton, Krizhevsky, Wang, 2011, Taigman, Yang, Ranzato, Wolf, 2014, Jaderberg, Simonyan, Zisserman, et al., 2015). Much of the early work on deep learning was performed using MNIST. Although good accuracies were achieved, the computer vision community did not take such work seriously because the images were so small. This, however, was the early days of deep learning (i.e., before 2012), and was a sign of things to come. This section describes an attempt to begin to reclaim some of the territory lost to deep learning.

Unlike most conventional pattern recognition approaches, the strategy adopted here is generative. Training involves learning independent models of the ten different digits in the training set, while testing involves fitting each model in turn to each image in the test set, and performing model comparison to assess which of the ten models better explains the data. The training stage involved learning $\hat{\mu },$ ${\hat{W}}^{a},$ ${\hat{W}}^{v}$ and $\hat{A}$ for each digit class. A similar strategy was previously adopted by Revow et al. (1996). From a probabilistic perspective, the probability of the *k*th label given an image (**f**) is

(52) $$P\left(M_{k}|f\right)=\frac{P(f,M_{k})}{P(f)}=\frac{\int_{z}P\left(f|z,M_{k}\right)p\left(z|M_{k}\right)dzP\left(M_{k}\right)}{\sum_{l=0}^{9}\int_{z}P\left(f|z,M_{l}\right)p\left(z|M_{l}\right)dzP\left(M_{l}\right)}$$

The above integrals are intractable, so are approximated. This was done by a “Laplace approximation”^6^ whereby the approximate distribution of **z** is given by

(53) $$q\left(z\right)=N\left(z|\hat{z},S^{-1}\right)$$

From this approximation, we can compute

(54) $$\int_{z}P\left(f,z|M\right)dz≃P\left(f,\hat{z}|M\right)\int_{z}\exp\left(-\frac{1}{2}{\left(z-\hat{z}\right)}^{T}S\left(z-\hat{z}\right)\right)dz=P\left(f,\hat{z}|M\right)\;{\left|S/\left(2\pi \right)\right|}^{1/2}$$

For each image (**f**), the mode ($\hat{z}$) of $p(f,z|M_{k})$ was computed (see Section 2.2.6) by

(55) $$\hat{z}=\underset{z}{\arg\min}\left(J(f,z,\mu ,{\hat{W}}^{a},{\hat{W}}^{v}\right)+\frac{1}{2}z^{T}\left(\lambda_{1}\hat{A}+\lambda_{2}{\left({\hat{W}}^{a}\right)}^{T}L^{a}{\hat{W}}^{a}+\lambda_{2}{\left({\hat{W}}^{v}\right)}^{T}L^{v}{\hat{W}}^{v}\right)z).$$

The Hessian of the objective function around this mode (2.2.6) was used to approximate the uncertainty ($S^{-1}$).

**Fig. 6:**
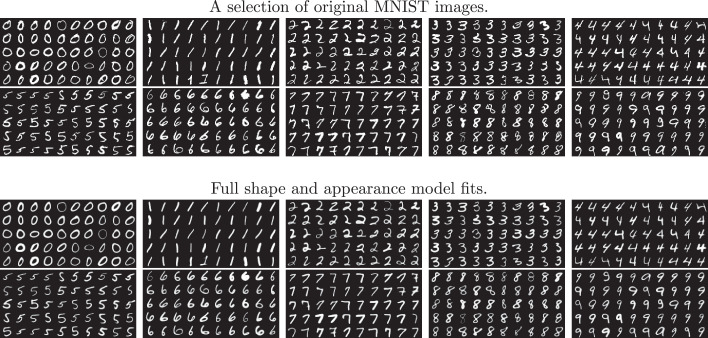
A random selection of digits from the first 10,000 MNIST training images, along with the model fit. In general, good alignment is achieved.

Training was done with different sized subsets (300, 500, 1000, 3000, 5000, 10,000, and all 60,000) of the MNIST training data, whereas testing was always done using the 10,000 test images. In each of the training subsets, the first of the images were always used, which generally leads to slightly different sized training sets for each of the digits. Example images, along with the fit from the models trained using the first 10,000 images, are shown in Fig. 6. Model fitting was run for 20 iterations, using a Bernoulli likelihood with K=16, $\nu_{0}=16,$ λ=[0.950.05], $\omega^{a}=\left[0.002\;0.2\;0\right],$ $\omega^{\mu }=N\left[10^{-7}\;10^{-5}\;0\right]$ and $\omega^{v}=\left[0.002\;0.02\;2\;0.2\;0.2\right]$.

When applied to medical images, machine learning can suffer from the curse of dimensionality. The number of pixels or voxels in each image (*M*) is often much greater than the number of labelled images (*N*) available for training. For MNIST, there are 60,000 training images, each containing 784 pixels, giving *N*/*M* ≃ 75. In contrast, even after down-sampling to a lower resolution, a 3D MRI scan contains in the order of 20,000,000 voxels. Achieving a similar *N*/*M* as for MNIST would require about 1.5 billion labelled images, which clearly is not feasible. For this reason, this section focuses on classification methods trained using smaller subsets of the MNIST training data. Accuracies are compared against those reported by Lee et al. (2015) for their Deeply Supervised Nets, which is a deep learning approach that performs close to state-of-the-art (for 2015), particularly for smaller training sets. Invariant scattering convolutional networks are also known to work well for smaller training sets, so some accuracies taken from Bruna and Mallat (2013) are also included in the comparison. We are not aware of more recent papers that assess the accuracy of deep learning using smaller training sets.

**Fig. 7:**
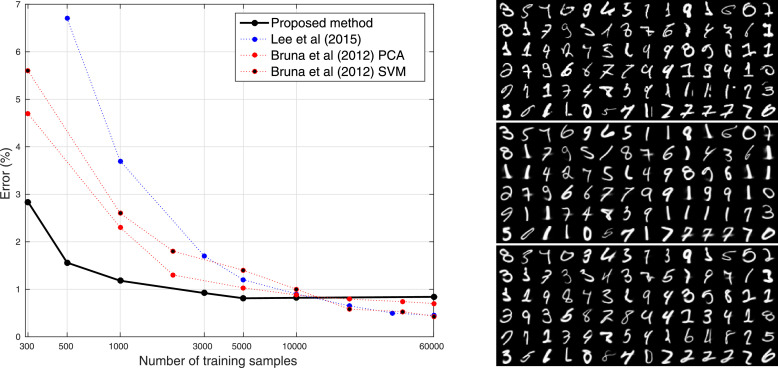
Left: Test errors from training the method using different sized subsets of the MNIST data (the error rate from random guessing would be 90%). Right: All the MNIST digits the method failed to correctly identify (after training with the full 60,000) are shown above. These are followed by the model fits for the true digit, and then the model fits for the incorrect guess (i.e., the one with the most model evidence). (For interpretation of the references to colour in this figure legend, the reader is referred to the web version of this article.)

Plots of error rate against training set size are shown in Fig. 7, along with the approximate error rates from Lee et al. (2015) and Bruna and Mallat (2013). The plot shows the proposed method to be more accurate than deep learning for smaller training sets, but it is less accurate when using the full training set, as the error rate plateaus to a value of about 0.85% for training set sizes of around 5000 onward. Visual assessment of the fits to the misclassified digits (Fig. 7) suggests that relatively few of the failures can be attributed to registration errors.

**Fig. 8:**
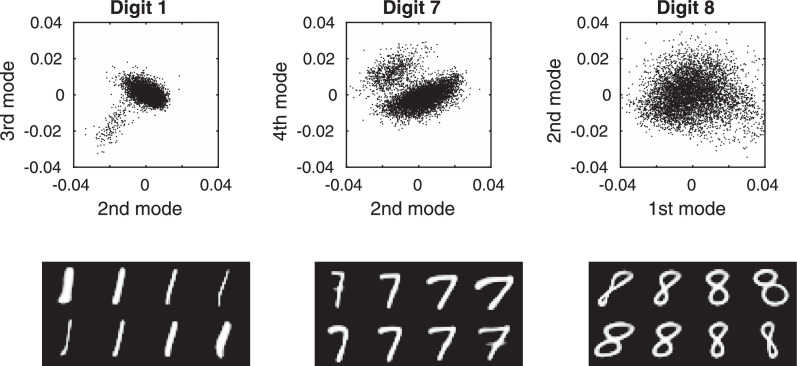
Illustration of the non-Gaussian distributions of the latent variables for some of the MNIST digits. Plots of selected latent variables are shown above, with the corresponding modes of variation shown below. Gaussian mixture models are likely to provide better models of variability than the current assumption of a single Gaussian distribution.

These experiments with MNIST suggest that one avenue of further work could be to elaborate on the simple multivariate Gaussian model for the distribution of latent variables. Although accuracies were relatively good for smaller training sets, the Gaussian assumptions meant that increasing the amount of training data beyond about 5000 examples did not bring any additional accuracy. One example of where the Gaussian distribution fails is when attempting to deal with sevens written either with or without a bar through them, which clearly requires some form of bimodal distribution to describe (see Fig. 8). One approach to achieving a more flexible model of the latent variable probability density would to use a Gaussian Mixture Model (GMM) (Cootes and Taylor, 1999). One of the aims of the Medical Informatics Platform of the HBP was to cluster patients into different sub-groups. In addition to possibly achieving greater accuracy, incorporating a GMM over the latent variables could also lead to this clustering goal being achieved.

### 3.3 Experiments with segmented MRI

Experiments were performed using 1913 T1-weighted MR images from the following datasets.

• The *IXI* dataset, which is available under the Creative Commons CC BY-SA 3.0 license from http://brain-development.org/ixi-dataset/. Information about scanner parameters and subject demographics are also available from the web site. Scans were collected on three different scanners using a variety of MR sequences. This work used only the 581 T1-weighted scans.
• The *OASIS Longitudinal* dataset is described in Marcus et al. (2010). The dataset contains longitudinal T1-weighted MRI scans of elderly subjects, some of whom had dementia. Only data from the first 82 subjects of this dataset were downloaded from http://www.oasis-brains.org/, and averages of the scans acquired at the first time point were used.
• The *COBRE* (Centre for Biomedical Research Excellence) dataset are available for download from http://fcon_1000.projects.nitrc.org/indi/retro/cobre.html under the Creative Commons CC BY-NC license. The dataset includes fMRI and T1-weighted scans of 72 patients with Schizophrenia and 74 healthy controls. Only the T1-weighted scans were used. Information about scanner parameters and subject demographics is available from the web site.
• The *ABIDE I* (Autism Brain Imaging Date Exchange) dataset was downloaded via http://fcon_1000.projects.nitrc.org/indi/abide/abide_I.html and is available under the Creative Commons CC BY-NC-SA license. There were scans from 1102 subjects, where 531 were individuals on the Autism Spectrum. Subjects were drawn from a wide age range and were scanned at 17 different sites around the world. All the T1-weighted scans were used, and these had a very wide range of image properties, resolutions and fields of view. For example, many of the scans did not cover the cerebellum.

The images were segmented using the algorithm in SPM12, which uses the approach described in Ashburner and Friston (2005), but with some additional modifications that are described in the appendices of Weiskopf et al. (2011) and Malone et al. (2015). Binary maps of grey and white matter were approximately aligned into ICBM152 space using a rigid-body transform obtained from a weighted Procrustes analysis (Gower, 1975) of the deformations estimated by the segmentation algorithm. These approximately aligned images have an isotropic resolution of 2 mm.

#### 3.3.1 2D experiments with segmented MRI

It is generally easier to visualise how an algorithm is working when it is run in 2D, rather than 3D. The examples here will be used to illustrate the behaviour of the algorithm under topological changes, when variability can not be modelled only via diffeomorphic deformations.

A single slice was extracted from the grey and white matter images of each of the 1913 subjects, and the joint shape and appearance model was fit to the data using the settings for categorical image data. This assumed that each voxel was a categorical variable indicating one of three tissue classes (grey and white matter, as well as background). Each 2D image was encoded by 100 latent variables (i.e. K=100). Eight iterations of the algorithm were used, with λ=[0.90.1], $\omega^{a}=\left[0.1\;16\;128\right],$ $\omega^{\mu }=N\left[0.0001\;0.01\;0.1\right],$ $\omega^{v}=\left[0.001\;0\;32\;0.25\;0.5\right]$ and $\nu_{0}=100$.

**Fig. 9:**
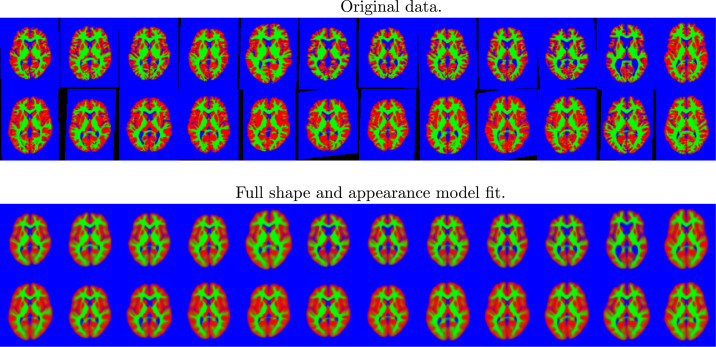
A random selection of the 2D brain image data, showing grey matter (red), white matter (green) and other (blue). Black regions indicate missing data. Below these is the model fit to the images. (For interpretation of the references to colour in this figure legend, the reader is referred to the web version of this article.)

**Fig. 10:**
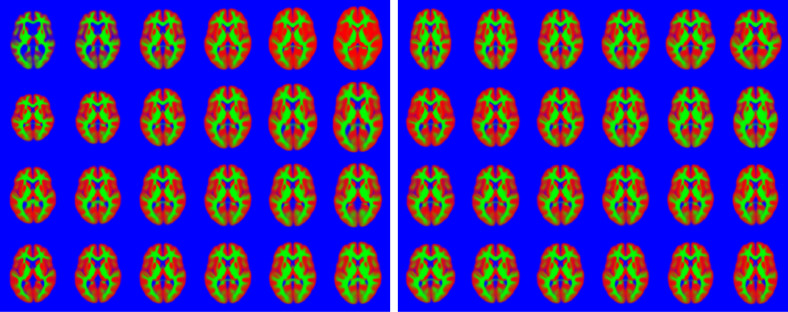
First eight (out of a total of 100) modes of variability found from the 2D brain image dataset, shown at −5, −3, −1, +1, +3 & +5 standard deviations. Note that these modes encode some topological changes, in addition to changes in shape. (For interpretation of the references to colour in this figure legend, the reader is referred to the web version of this article.)

Some model fits are shown in Fig. 9, and the principal modes of variability are shown in Fig. 10, which shows that these images are reasonably well modelled. Note that the topology of the images may differ, which (by definition^7^) is not something that can be modelled by diffeomorphisms alone. The inclusion of the appearance model allows these topology differences to be better captured.

#### 3.3.2 Imputing missing data

The ability to elegantly handle missing data is a useful requirement for mining hospital scans. These often have limited fields of view, and may miss out parts of the brain that are present in other images. The objective here is to demonstrate that a reasonable image factorisation can be learned, even when some images in the dataset may not have full organ coverage.

This experiment used the same slice through the data as above, and a rectangle covering 25% of the area of the images was placed randomly in each and every image of the training set (wrapping around at the edge of the field of view), and the intensities within these rectangles set to NaN (“not a number” in the IEEE 754 floating-point standard). The algorithm was trained, using the same settings as described previously, on the these modified images. Although imputed missing values may not be explicitly required, they do provide a useful illustration of how well the model works in less than ideal situations. Fig. 12 shows a selection of the images with regions set to NaN, and the same images with the missing values predicted by the algorithm.

The ability to handle missing data allows cross-validation to be used to determine the accuracy of a model, and how well it generalises. In addition to the joint shape and appearance model, this work also allows simplified versions to be fitted that involve only shape (i.e., not using **W**^*a*^, as in Zhang and Fletcher (2015)) or in a form that varies only the appearance (i.e. not using **W**^*v*^). This work also includes a version where different sets of latent variables control the shape and appearance. Here, there were 30 variables to control appearance $K^{a}=30$ in (Eq. (13)), and 70 to control shape ($K^{v}=70$ in (Eq. (14))). The aim was to compare the four models by assessing how well they are able to predict data that was unavailable to the model during fitting. This gives us ground truth with which to compare the models’ predictions, and is essentially a form of cross-validation procedure. Accuracy was measured by the log-likelihood of the ground truth data, which was computed only for pixels that the models did not have access to during training.

The results of the cross-validation are shown in Fig. 13, and show that the two models that combine both shape and appearance have greater predictive validity than either the shape or appearance models alone. To clarify the general pattern, the log-likelihoods of each patch were also plotted after subtracting their mean log-likelihood over all model configurations. Although the difference was small, the best results were from the model where each latent variable controls both shape and appearance, rather than when they are controlled separately ($p<10^{-5}$ from a paired *t*-test).

Changes to hyper-parameter settings, etc. may improve accuracies further. The effects of changing *ω^a^* and *ω^v^* were assessed by running a similar comparison using the model where the same latent variables control both shape and appearance. The hyperparameter settings were varied over two orders of magnitude by scaling the previously used settings by 0.1, 1 and 10. In addition, the settings for *ω^μ^* were decreased by a factor of 100. Results are shown in Fig. 14, and gave the best accuracies with $\omega^{a}=\left[0.01\;1.6\;12.8\right]$ and $\omega^{v}=\left[0.001\;0\;32\;0.25\;0.5\right]$. Using the smaller *ω^μ^* made an insignificant difference to the average log likelihoods (result not shown). Paired t tests between all pairs of comparisons showed that the choice of hyperparameter settings plays an important role. A similar comparison could also be made by varying other hyper-parameter settings.

#### 3.3.3 3D experiments with segmented MRI

The aim of this section was to apply the method to a large set of 3D images, and use the resulting latent variables as features for pattern recognition. For this, a version of the model was used whereby some latent variables controlled appearance, whereas others controlled shape. The motivation for this was that it allows the different types of features to be differentially weighted when they are used to make predictions.

The algorithm was run on the full 3D dataset, using 70 variables to control shape ($K^{v}=70$) and 30 to control appearance ($K^{a}=30$). Eight iterations were used, with λ=[11], $\omega^{a}=\left[0.01\;1\;50\right],$ $\omega^{\mu }=N\left[0.00001\;0.01\;0.1\right]$ and $\omega^{v}=\left[0.001\;0\;10\;0.1\;0.2\right]$. Slice 40 of the resulting mean image is shown in Fig. 15, alongside the mean from one of the 2D experiments. Note that the mean from the 2D model is slightly crisper than that from the one in 3D. The main reason for this is simply that it is a 3D fit, so that there is a great deal more variability to explain. Achieving a similar quality of fit for the full 3D data, as was achieved for the 2D data, would require in the order of 1000 (100^3/2^) variables.

**Fig. 11:**
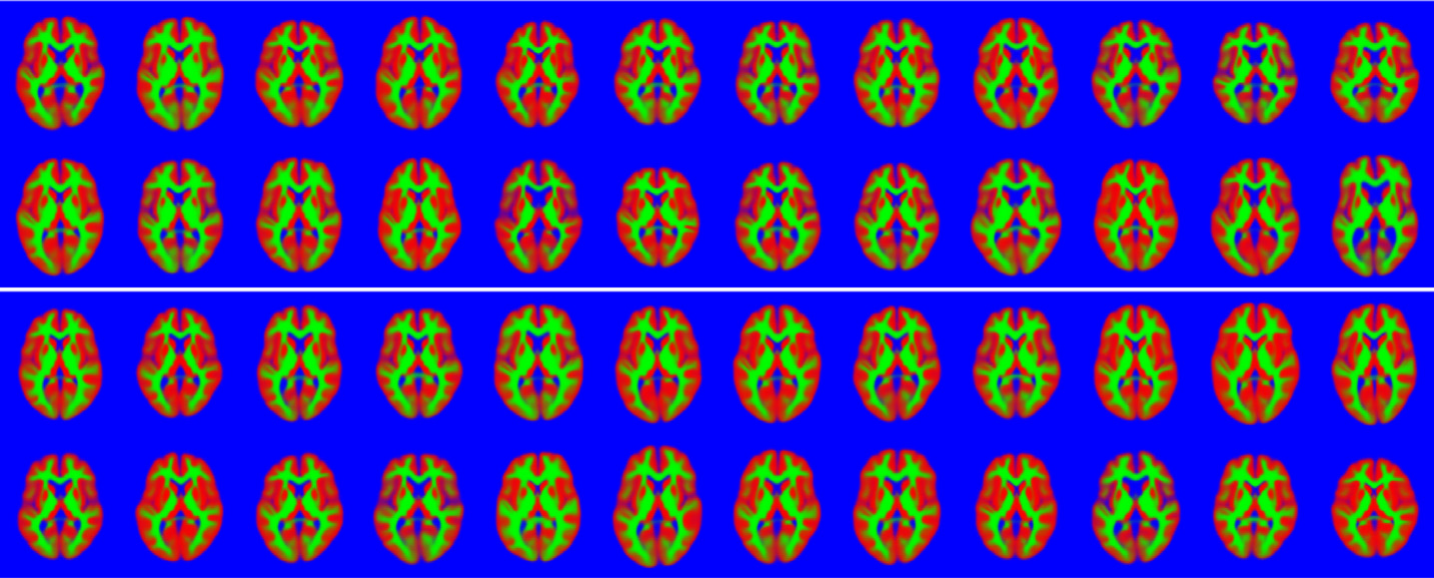
Randomly generated slice through brain images. These images were constructed by using randomly assigned latent variables. Note that the top set of images uses the same random variables as the bottom set, except they are multiplied by −1. This means that one set is a sort of “opposite” of the other. For example, if a brain in the upper set has large ventricles, then the corresponding brain in the lower set will have small ventricles. (For interpretation of the references to colour in this figure legend, the reader is referred to the web version of this article.)

**Fig. 12:**
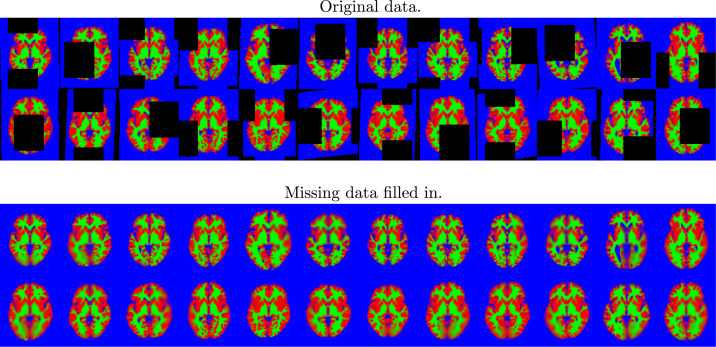
A random selection of the 2D brain image data showing the location of missing data. The attempt to fill in the missing information is shown below. These may be compared against the original images shown in Fig. 9. (For interpretation of the references to colour in this figure legend, the reader is referred to the web version of this article.)

**Fig. 13:**
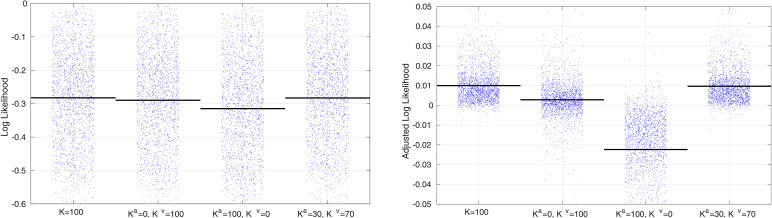
Cross-validation accuracy measures based on predicting the left-out patches of the images using different model configurations. The blue dots show the mean value for each of the 1913 images, whereas the horizontal bars show the mean values overall. The plot on the left shows mean log-likelihoods over the pixels in each patch, wheres the plot on the right shows the log-likelihoods after subtracting the mean – over model configurations – for each patch.

**Fig. 14:**
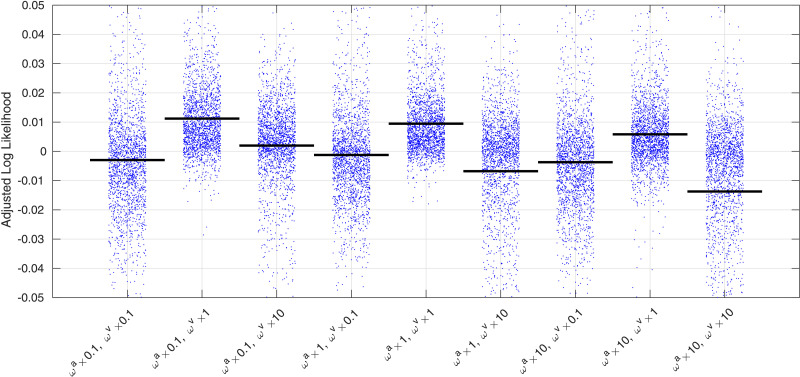
Cross-validation accuracy measures based on predicting the left-out patches of the images using different hyper-parameter settings. The blue dots show the mean value for each of the 1913 images, whereas the horizontal bars show the mean values overall. Accuracy measures are mean log-likelihoods (over voxels), after adjustment.

**Fig. 15:**
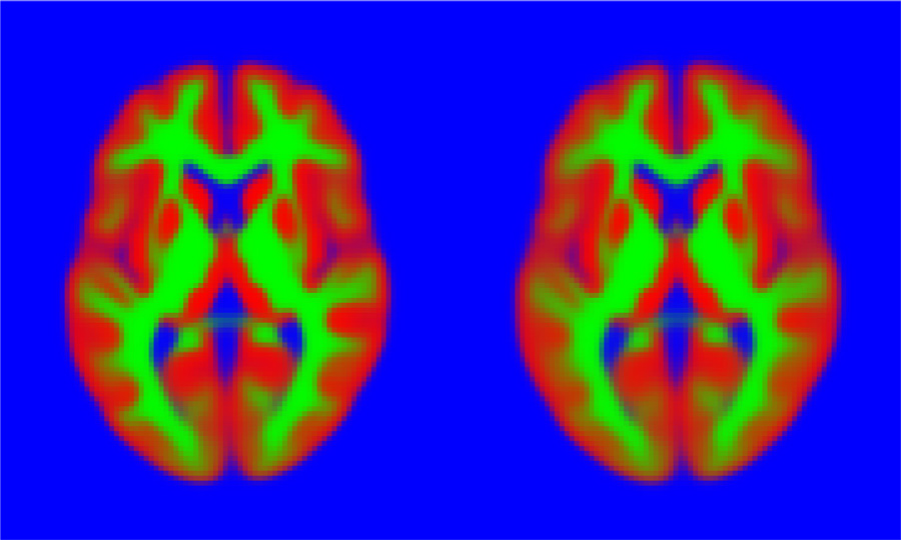
An illustration of the mean images from the 2D and 3D experiments (after Softmax). Left: The mean image from the 2D experiments (c.f. Figs. 9 and 10). Right: Slice 40 of the mean image from the 3D experiment. (For interpretation of the references to colour in this figure legend, the reader is referred to the web version of this article.)

**Fig. 16:**
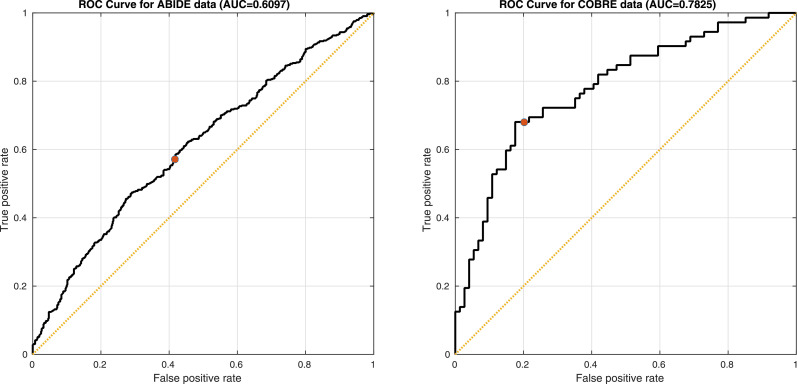
ROC curves from five-fold cross-validation accuracies from the ABIDE and COBRE data. Red dots show the point on the curve where the classification gives probabilities of 0.5.

The main objective of this work is to extract a small number of features from sets of anatomical medical images, which are effective for machine learning applications. Here, a five-fold cross-validation is used to assess the effectiveness of these features. Machine learning used a linear Gaussian process classification procedure, which is essentially equivalent to a Bayesian approach to logistic regression. The implementation was based on the method for binary classification using expectation propagation described in Rasmussen and Williams (2006). For the COBRE dataset, classification involved separating controls from patients with schizophrenia. Similarly, the analysis of the ABIDE dataset involved identifying those subjects on the autism spectrum, with features orthogonalised with respect to the different sites. Classification involved three hyper-parameters, which weighted the contributions from shape features, appearance features and a constant offset. Resulting ROC curves are shown in Fig. 16.

For ABIDE, the accuracy and 95% confidence interval was 57.6 ± 2.9%. While this is not especially high, it is close to the accuracy reported by others who have applied machine learning to the T1-weighted scans. Most previous works (Haar, Berman, Behrmann, Dinstein, 2014, Katuwal, Cahill, Baum, Michael, 2015, Ghiassian, Greiner, Jin, Brown, 2016) have reported their best classification accuracies of around 60% when using the same dataset. Results are roughly comparable with some of the accuracies obtained by Monté-Rubio et al. (2018) or Demirhan (2018). Those papers reported multiple accuracies, so it would be difficult to choose a single accuracy with which to compare.

The accuracy achieved for the COBRE dataset was 74.7 ± 7.1%, which is similar to the 69.7% accuracy reported by Cabral et al. (2016) using COBRE, and was roughly comparable with many of the accuracies obtained by Monté-Rubio et al. (2018) or Demirhan (2018). Others have used other datasets of T1-weighted scans for identifying patients with schizophrenia. Nieuwenhuis et al. (2012) achieved 71.4% and Ma et al. (2018) achieved 75.8% accuracy for separating controls from subjects with schizophrenia, but using larger datasets.

Anatomical T1-weighted MRI is unlikely to be the most useful type of data for assessing psychiatric disorders, and better classification accuracies have been achieved using other modalities, such as fMRI (Silva et al., 2014). We note that some other papers have reported much higher accuracies using the COBRE dataset, but many of these works made use of manual annotations or may not have kept a strict separation between testing and training data.

### 3.4 Experiments with head and neck

Most conventional image registration algorithms involve some form of local optimisation, and are therefore susceptible to getting caught in local optima. Good initialisation can help avoid such optima. This is often achieved by registering via a rigid or affine transform, which captures some of the main modes of shape variability. However, this does not capture the main ways that biological structures may vary in shape, and it may be possible to do better. In this section, we examine how suited the proposed model is to this task by comparing “groupwise” registrations initialised with affine transforms versus those initialised using the proposed method. The Ants software^8^ (Avants et al., 2014) was used for this, as it is widely accepted to be an effective image registration package.

The data were the 581 T1-weighted scans from the IXI dataset, which were approximately rigidly aligned and downsampled to an isotropic resolution of 1.75 mm. The resulting images all had dimensions of 103 × 150 × 155 with a field of view that covered both head and neck, and were scaled to have maximum value of 1.0. Approximately binary masks of the brains within the original T1-weighted scans were extracted using the segmentation module (Ashburner and Friston, 2005) of the SPM12 software^9^, and these were also transformed in the same way.

1. For the case where Ants was initialised via affine transforms, registration was run serially in 3D using one of the scripts released with the software (Avants, Yushkevich, Pluta, Minkoff, Korczykowski, Detre, Gee, 2010, Avants, Tustison, Song, Cook, Klein, Gee, 2011). The script first corrected the images for smooth variations in intensity nonuniformity using N4 (Tustison et al., 2010), and the actual registration minimised the local correlation coefficients via a greedy gradient descent.
  antsMultivariateTemplateConstruction.sh -d3 -c0 -o ants *.nii
  The warps generated by Ants were applied to all the brain masks to bring them into a common space.
2. The proposed method was also run on the data, using 20 iterations with the Gaussian noise model, $K^{a}=4,$ $K^{v}=60,$ $\omega^{v}=\left[0.01\;0\;10\;1\;2\right],$ $\omega^{a}=\left[100\;1000\;0\right],$ $\omega^{\mu }=\left[0.01\;10\;0\right],$ $\nu_{0}=140$ and λ=[9.50.5]. The resulting parameter estimates were then used to warp all the images to approximately match the mean, before the alignment was refined further by applying Ants to these warped images. Warps generated by the proposed model were composed with those generated by Ants, and the result was used to warp all the brain masks into a common space.

The mean (***μ***) of all the binarised aligned mask images was computed and the following Jaccard and binomial log-likelihood overlap measure derived for each (**b**) of them.

(56) $$J(\mu ,b)=\frac{\sum_{m=1}^{M}\left(\left(\mu_{m}>\frac{1}{2}\right)\wedge b_{m}\right)}{\sum_{m=1}^{M}\left(\left(\mu_{m}>\frac{1}{2}\right)\vee b_{m}\right)}L(\mu ,b)=\frac{1}{M}\sum_{m=1}^{M}\left(b_{m}\log_{2}\mu_{m}+\left(1-b_{m}\right)\log_{2}\left(1-\mu_{m}\right)\right)$$

**Fig. 17:**
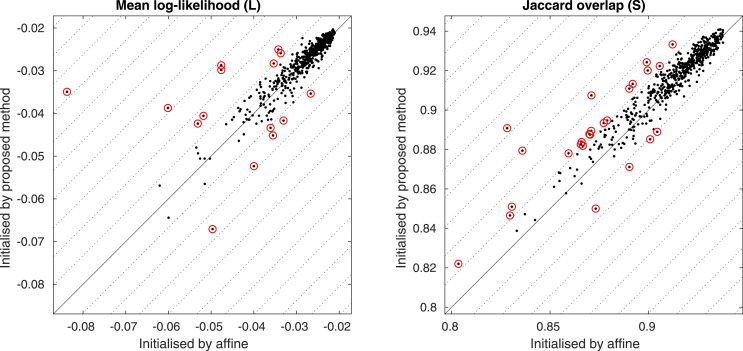
Overlap measures from the two registration approaches. Diagonal lines are spaced two standard deviations apart. Circled points indicate outliers of more than two standard deviations.

We note that these measures reflect overlap of “spatially normalised” images, which is what typically interests many users of registration software.^10^The resulting overlap measures are shown in Fig. 17, and are mostly similar between the two approaches. However, the pattern of outliers (more outliers in the top left than in the bottom right) suggests that using the proposed approach to initialise registration leads to slightly more robust alignment. An analysis based on the Jacard overlap, counting outliers beyond 2 standard deviations, would show a clear benefit of the proposed method, but the pattern is less certain when the log-likelihood measures are also considered. Because the numbers of outliers are relatively small, it is difficult to draw firm statistical conclusions.

**Fig. 18:**
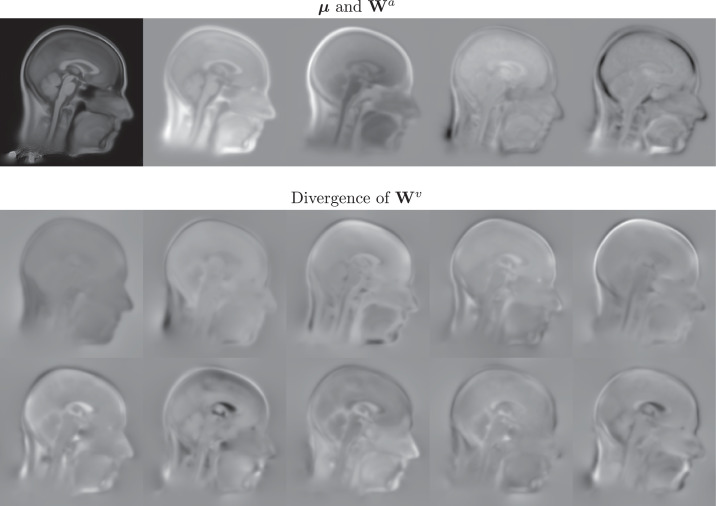
Mid-sagittal slice through the basis functions. The mean (***μ***) and four appearance basis functions (**W**^*a*^) are shown above, while the divergences of the first 10 shape basis functions (**W**^*v*^) are shown below.

Fig. 18 shows the mid-sagittal slice through a selection of the basis functions estimated by the proposed model. The four appearance basis functions were intended to capture variability across scanners, plus a few other sources of signal intensity variability such as that of bone marrow in the skull. Rather than the individual components of the shape basis functions, their divergence is shown instead in Fig. 18. These divergence maps encode expansion or contraction within the diffeomorphic deformations. The first of these is mostly concerned with overall head size (and suggest that larger heads are associated with greater bulk at the back of the neck), whereas the second and third components appear to mostly capture variability related to the amount of body fat – particularly in the neck. Other shape components encode neck angulation and various other aspects of head shape variability. The proposed model was run with only 60 shape components because the intention was to assess its utility for capturing the main modes of variability, as a precursor to the finer alignment.

## 4 Discussion

This work presents a very general generative framework that may have widespread use within the medical imaging community, particularly for those situations where conventional image registration approaches are more likely to fail. Because of its generality, the model we presented should provide a good starting point for a number of avenues of further development.

Most image analysis applications have a number of settings to be tuned, and the current approach is no exception. Although this tuning is rarely discussed in papers, the settings can have quite a large impact on any results. We propose that a cross-validation strategy, as shown in Section 3.3.2, could be used for this. The approach taken in this work is simply to treat the construct as a model of the data, and to assess it according to how well it describes and predicts the observations. This work does not consider identifiability issues relating to how well it can separately estimate shape information versus appearance information.

Additional attention is the setting of *λ*_1_ and *λ*_2_ may be needed. From the perspective of the underlying generative model used, these settings should ideally sum to 1. In practice however, greater regularisation ($\lambda_{1}+\lambda_{2}>1$) is required in order to achieve good results. A plausible explanation for this would be that assumptions of i.i.d. noise are not generally met, so a “virtual decimation factor”, which accounts for correlations among residuals, may need to be accounted for Groves et al. (2011). The fact that the approach is not fully Bayesian (i.e., it only makes point estimates of many parameters and latent variables, rather than properly accounting for their uncertainty) may be another reason why additional regularisation is needed.

One aspect of the presented approach that is slightly unconventional is the scaling by *N* of **L**^*v*^ and **L**^*a*^ in (Eqs. (7)) and (8). Normally when constructing probabilistic generative models, the priors should not be adjusted according to how much data is available. An exception was made here because it has the effect of pushing the solution towards the basis functions encoding unit variance, rather than a variance that scales with *N*, with a corresponding decrease in the variance of the latent variables. In terms of the overall model fit, this only influences the behaviour of the prior $p\left(A\right)=W_{K}\left(A|I/\nu_{0},\nu_{0}\right),$ which in turn influences the variance of the latent variables. Without this Wishart prior, the scaling by *N* could have been omitted without affecting the overall model fits. An alternative strategy could have involved constraining the basis functions such that ${\left(W^{v}\right)}^{T}L^{v}W^{v}=I$.

Another limitation of our proposed shape and appearance model is that it assumes that appearance and shape evolve separately, such that the appearance changes are added to the mean, and then the results are deformed to match the individual images. It may be possible to achieve slightly improved results by incorporating a metamorphosis approach (Trouvé and Younes, 2005), which considers that shape and appearance evolve simultaneously. It is currently unclear whether the benefits from this type of elegant approach could bring enough practical benefit to make it worthwhile. Appearance changes and deformations are both typically relatively small, so an improvement in how the interaction between the two types of variability are handled seems unlikely to make an easily discernible difference.

There are a number of directions in which the current work could be extended. One avenue would be to allow some shape variability beyond what can be encoded by the first few eigenmodes. For example, Balbastre et al. (2018) combined the eigenmode representation with a model of additional shape variability, giving a framework that is conceptually related to that of Allassonnière et al. (2007), as this allows a covariance matrix over velocity fields to be defined and optimised.

The framework would also generalise further for handling paired or multi-view data, which could add a degree of supervision to the method. There have been a number of publications on generating age- or gender-specific templates, or on geodesic regression approaches (Niethammer, Huang, Vialard, 2011, Fletcher, 2013) for modelling trajectories of ageing. Concepts from joint matrix factorisation approaches, such as canonical correlation analysis (Bach, Jordan, 2005, Klami, Virtanen, Kaski, 2013), could be integrated into the current work, and these could be used to allow the model fitting to be informed by age, gender, disease status etc.

## Declaration of competing interest

The authors have no conflicts of interest to declare.

## Appendix A Notation

In most of this paper, matrices are written in bold upper-case (e.g., **W**^*a*^, **Z**, etc). In the computations, images are treated as vectors. These are written as lower-case bold, which includes the notation for individual columns of various matrices (e.g., $w_{k}^{a}$ denotes the *k*th column of **W**^*a*^, **z**_*n*_ denotes the *n*th column of **Z**, etc). Scalars are written in italic, with dimensions in upper-case. Estimates or expectations of parameters are written with a circumflex (e.g., $\hat{Z}$). Collections of vectors may be conceptualised as matrices, so are written in bold-upper-case (e.g., **G**^*a*^, where individual vectors are $g_{k}^{a}$). Collections of matrices are written in “mathcal” font (e.g., $H^{a},$ where individual matrices are $H_{kk}^{a}$). The matrix transpose operation is denoted by the “^*T*^” superscript (as in **Ψ**^*T*^). Creating a diagonal matrix from a vector (as in diag(exp **q**)), as well as treating the diagonal elements of a matrix as a vector (as in diag(**Q**)) are both denoted by “diag”. The trace of a matrix (sum of diagonal elements) is denoted by “*Tr*”.

This paper mixes both discrete and continuous representations of the same objects. For the discrete case, where a velocity field is treated as a vector, it is denoted by **v**_*n*_. Alternatively, the same object may be treated as a continuous 3D vector field, where it is denoted by *v_n_*.

In addition, deformations may be treated as discrete or continuous. Within the continuous setting, warping an entire image by a diffeomorphism *ψ* may be denoted by $a^{\prime }=a\left(\psi \right)$. In the discrete setting, this resampling may be conceptualised as a matrix multiplication, where a very large sparse matrix **Ψ** encodes the same deformation (and associated trilinear interpolation), such that $a^{\prime }=\Psi a$. The transpose of this matrix can be used to perform a push-forward operation, which is frequently used in this work and which we denote by $f^{\prime }=\Psi^{T}f$.

Sometimes, gradients of an image are required. In 3D, the three components of the spatial gradient of **a** are denoted by ∇_1_**a**, ∇_2_**a** and ∇_3_**a**.

## Footnotes

2 See https://en.wikipedia.org/wiki/Loewner_order.

3 https://github.com/WTCN-computational-anatomy-group/Shape-Appearance-Model.

4 http://yann.lecun.com/exdb/mnist/.

5 This was said by the late David MacKay (MacKay, 2003) in relation to the success of kernel methods, such as support-vector machines or Gaussian processes, which, at the time, were replacing neural networks in practical applications.

6 For a textbook explanation of Bayesian approaches, including the Laplace approximation, see MacKay (2003), Bishop et al. (2006) or Murphy (2012).

7 Topology is concerned with properties that are preserved following diffeomorphic deformations (see https://en.wikipedia.org/wiki/Topology).

8 https://github.com/ANTsX/ANTs.

9 https://www.fil.ion.ucl.ac.uk/spm/software/spm12/.

10 From a modelling perspective, the overlaps would have been better computed by warping the mean to match each individual image.

## Footnotes

1 Note that the latent precision matrix **A** should not be confused with the appearance variables **a**_*n*_, which were introduced earlier. Hopefully, the context in which they are used should be enough to prevent any confusion.
